# Tomographic and volcanotectonic control on the 2021–2023 Fagradalsfjall eruptions, Iceland

**DOI:** 10.1038/s41598-025-95169-6

**Published:** 2025-05-12

**Authors:** Alex Hobé, Mohsen Bazargan, Burcu Selek, Ari Tryggvason, Emmanuel Alofe, Agust Gudmundsson

**Affiliations:** 1 Department of Earth Sciences, Uppsala University, Villavägen 16, Uppsala, 752 36 Sweden; 2 Department of Earth Sciences, Royal Holloway University of London, Queen’s Building, Egham, TW20 0EX UK

**Keywords:** Natural hazards, Seismology, Volcanology, Geophysics

## Abstract

Shortly before the 2021 Fagradalsfjall eruption tomographic images indicated a large high V_p_/V_s_ anomaly with a top at about 9 km depth directly below the ongoing seismic swarm. Using volcanotectonic principles we interpret this anomaly as part of the roof of a magma reservoir which, furthermore, ruptured on 24 February 2021. The roof rupture resulted in an injected dike-segment propagating vertically at an average rate of about 0.2 m s^− 1^ until it became arrested beneath a stress barrier at ~ 2 km depth, from where the dike-segment propagated laterally. Further magma injections took place, following largely the path of the first dike-segment, resulting in part of the dike reaching outside the stress barrier and propagating vertically as a tiny ‘finger’ to form a 180-m-long disconnected fissure at the surface on 19 March 2021. The estimated maximum length of the arrested dike is about 9 km, its height 7 km, and its thickness 4 m – in excellent agreement with direct field measurements of basaltic dikes in Iceland. Seismicity also indicates that the same dike path was partly used during the 2022 and 2023 eruptions, in agreement with the location of the resulting volcanic fissures, resulting in the formation of a multiple feeder-dike.

**Supplementary Information:**

The online version contains supplementary material available at 10.1038/s41598-025-95169-6.

## Introduction

**Fig. 1:**
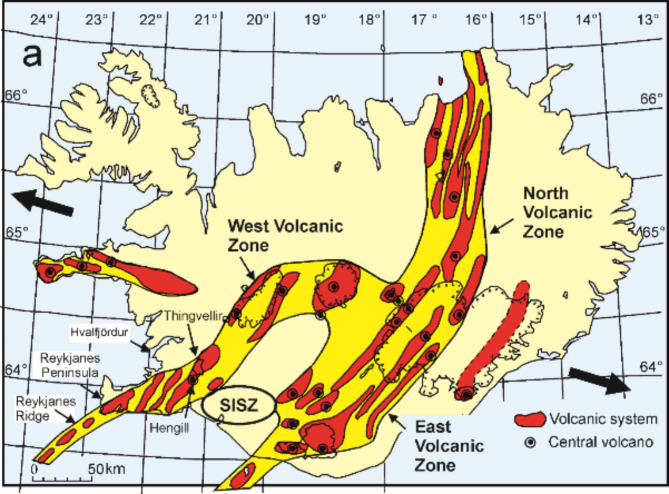

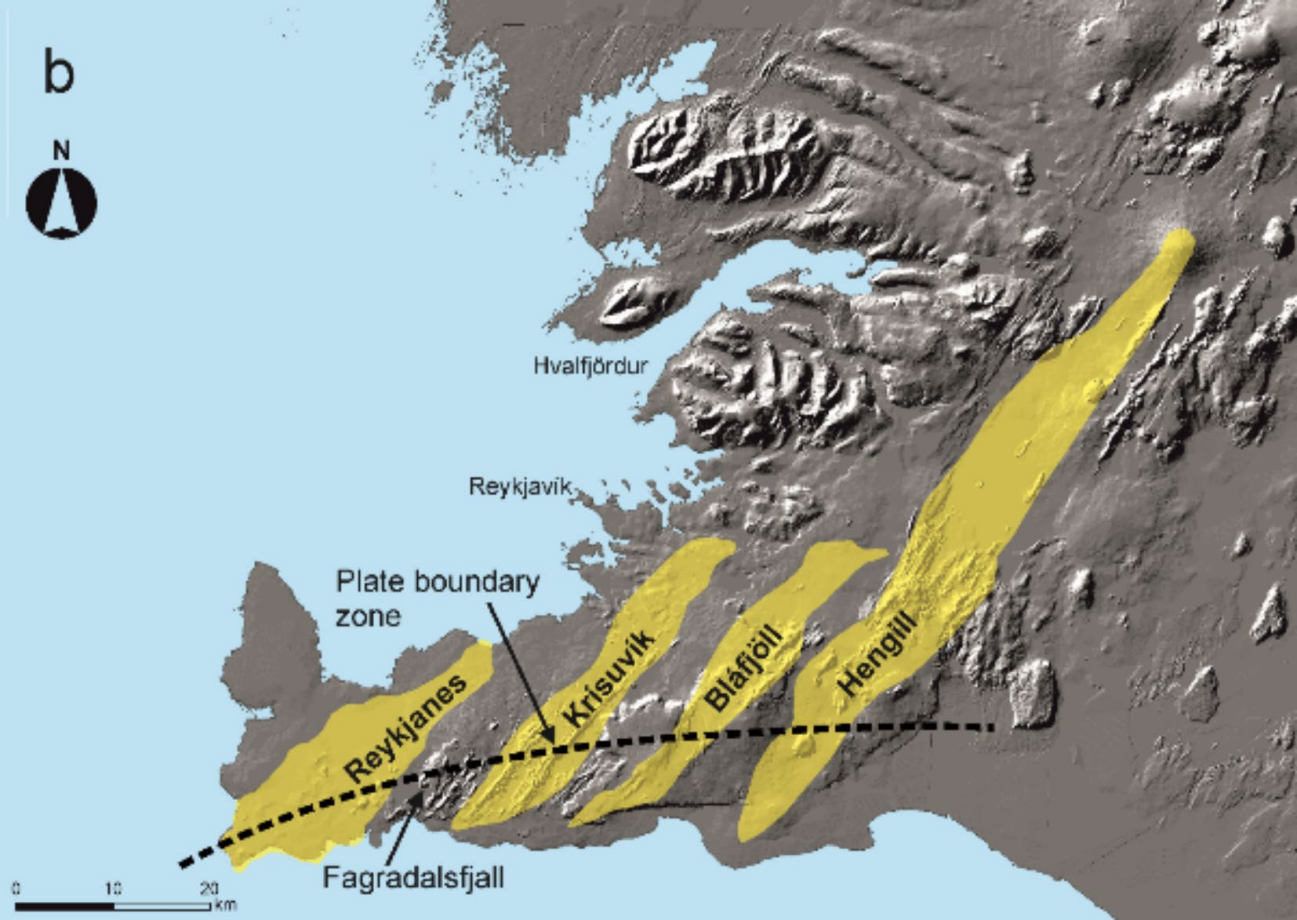
(**a**) Volcanic zones (bright yellow colour) and systems (red) of Iceland active during Holocene (the past 11,700 years). The systems are mostly zones or swarms of volcanoes formed in single eruptions, such as volcanic fissures (crater rows), lava shields, and various types of single craters, as well as tectonic fractures. Here we show 28 volcanic systems, but their exact number and geometries vary somewhat depending on the criteria used to define them. Most volcanic systems develop central volcanoes (stratovolcanoes, collapse calderas; indicated by encircled black dots) supplied with magma from shallow magma chambers. The Hengill Volcanic System (whose northern part is the Thingvellir Graben, indicated) has a central volcano, Hengill. The systems on the Reykjanes Peninsula proper have neither a central volcano nor a long-lived shallow chamber. Also indicated are the northernmost part of the Reykjanes Ridge, the fjord Hvalfjördur (where deeply eroded volcanic systems similar to those on the Reykjanes Peninsula occur), and the South Iceland Seismic Zone (SISZ). The thick black arrows indicate the average direction of the spreading in Iceland. Modified from^8^ and prepared with Inkscape version 1.3.2 (https://inkscape.org/). (**b**). Main volcanic systems on the Reykjanes Peninsula are Reykjanes, Krísuvík, and Bláfjöll. The system of Hengill extends into the easternmost part of the peninsula, but is really a part of the West Volcanic Zone (Fig. 1a). Background digital elevation model is the 2-m ÍslandsDEM from Landmælingar Íslands (National Land Survey of Iceland). The broken line shows the highly oblique plate boundary zone where most of the earthquakes on the peninsula occur. Also indicated is the location of the capital Reykjavik and Fagradalsfjal, as well as the fjord Hvalfjördur. The information in the greyscale corresponds to the shaded topography. Modified from^8^ and prepared with Inkscape version 1.3.2 (https://inkscape.org/).

**Fig. 2:**
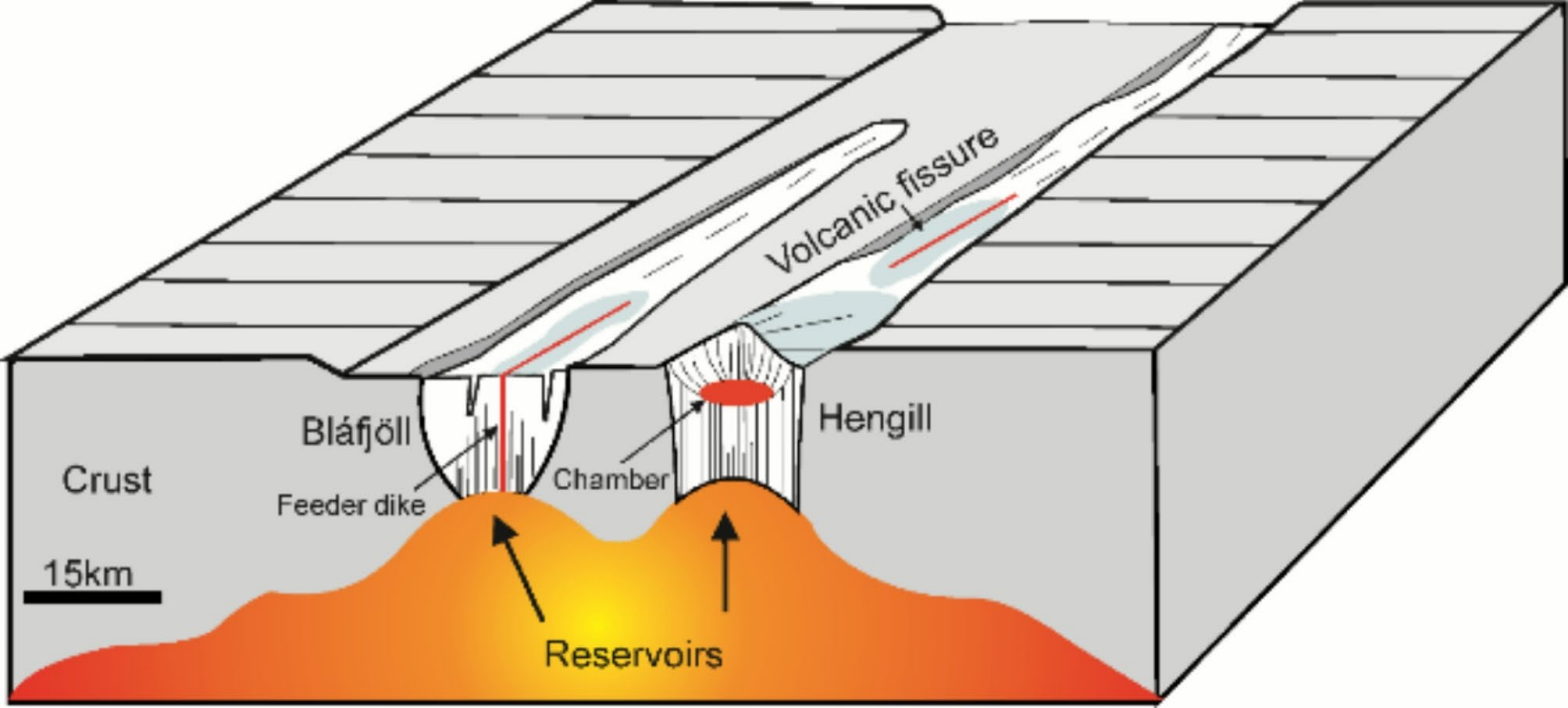
Schematic illustration of the internal structure of volcanic systems on and close to the Reykjanes Peninsula and elsewhere in the rift zone of Iceland^1^. In this illustration, the system on the right is similar to that of the Hengill Volcanic System, while the system on the left is similar to that of the Bláfjöll, as well as the other systems on the Reykjanes Peninsula (Fig. 1b). The Hengill System has a shallow magma chamber and also a deep-seated source reservoir, that is, a double magma chamber. The shallow chamber injects radial dikes and inclined sheets. By contrast, the Bláfjöll System (and the other systems on the peninsula) has only a deep-seated reservoir; here one feeder-dike is indicated as being injected from the reservoir, similar to that in the 2021–2023 Fagradalsfjall eruptions.

Prior to the Fagradalsfjall eruption on 19 March 2021 there had been no eruption on the Reykjanes Peninsula in Southwest Iceland for 781 years and in the Fagradalsfjall volcano for about 3000 years (Fig. 1). Deep-seated reservoirs were proposed beneath the volcanic systems of Iceland^1^, including those on the Reykjanes Peninsula, in the 1980s (Fig. 2). Many such reservoirs have since been detected beneath active volcanic systems in Iceland^2–4^, but none beneath the systems on the Reykjanes Peninsula (Fig. 1b) until shortly before the present eruptions.

Many active and fossil volcanic systems in Iceland and elsewhere have a major polygenetic volcano, a central volcano, located somewhere near the centre of the system (Fig. 1a). A central volcano is supplied with magma from a shallow magma chamber^5^ whose roof is normally at crustal depths of less than 6 km. Magma injections from such chambers take the form of (1) vertical dikes, many of which propagate primarily laterally (radially) from the chamber, (2) inclined sheets (cone sheets), and (3) sills. Most observed dike-induced earthquake swarms in the past decades have been interpreted as being due to lateral dike propagation^6,7^, which requires a shallow magma chamber of a suitable shape from which the dike can be injected into the crustal segment hosting the chamber^5^.

Neither long-lived shallow magma chambers nor central (polygenetic) volcanoes, however, exist on the Reykjanes Peninsula proper. The closest example of both to the peninsula is the Hengill Central Volcano^8^ in the West Volcanic Zone (Fig. 1). There, the existence of a shallow chamber is indicated, for example, by the appearance of silicic rocks which are mostly generated in shallow magma chambers^5^. Silicic extrusive rocks are absent from the Reykjanes Peninsula. In the absence of shallow magma chambers, dike injections on the Reykjanes Peninsula must be largely through vertical propagation from the deep-seated reservoirs (Fig. 2) – but until the 2021 Fagradalsfjall eruption there was no seismic evidence for such dike propagation.

On the Reykjanes Peninsula, a continuation of the oblique-spreading Reykjanes Ridge (Fig. 1a), the overall ENE strike of the plate-boundary zone is oblique to the orientations of the main volcanic systems that are generally NE-striking (Fig. 1b). Both the plate boundary and the volcanic systems are oblique to the spreading vector, whose average rate is about 1.8 cm yr^− 1^ in the direction of N105°E (Fig. 1a). While there are normal faults in the plate-boundary zone, it is characterised by conjugate strike-slip faults, where the NNE-striking faults are mostly dextral and the ENE-striking faults mostly sinistral^8^. The strike-slip faults are frequently seismically active and commonly independent of any volcanic activity^8^.

The three main volcanic systems on the Reykjanes Peninsula are Reykjanes, Krísuvík, and Bláfjöll, while part of the Hengill System extends into the easternmost part of the peninsula (Fig. 1b). The surface parts of the volcanic systems are characterised by tension fractures, normal faults, volcanic fissures, and lava shields. Studies in deeply eroded volcanic systems in Iceland show that tension fractures are confined to their uppermost several hundred metres. The frequency of normal faults decreases with depth, while that of dikes increases^9^.

Fagradalsfjall, of Pleistocene age, has been suggested as the centre of a separate volcanic system^10^ - the 4th system on the peninsula proper (Fig. 1b) - whose main Holocene structures are lava shields. The proposed system is mostly located within the plate-boundary zone (Fig. 1b), so that strike-slip faults are common but there is no swarm of normal faults and tension fractures. The existence of Fagradalsfjall as a separate system is debated, however. Also, because most of the Reykjanes Peninsula is volcanically active, magma might be expected to be available beneath Fagradalsfjall, occasionally at least, even if it is just the marginal part of the Reykjanes System.

The course of the Fagradalsfjall eruption that began on 19 March 2021, and its products, is described in detail elsewhere^11–13^, and will not be repeated here. The principal aim of this paper is to combine a tomographic model, relocated seismicity, and volcanotectonic principles to put constraints on and quantify the physical and geological parameters that controlled the three eruptions in 2021–2023, with a focus on the 2021 eruption. The parameters include (1) the location, formation, and rupture of the source magma reservoir, (2) the dike injection, dike dimensions, and its rate of propagation, and (3) the magmatic overpressure (driving pressure) and velocity in the eventual feeder-dike segment.

## Results

Our results are reported as follows. First we describe the high Vp/Vs anomaly below Fagradalsfjall identified in a tomographic study. Then we explain mechanically how a magma reservoir could have formed where the anomaly is located. Next we describe how magmatic excess pressure in the reservoir roof may have contributed to the unrest that preceded the eruption. The magmatic excess pressure is then put into context of reservoir rupture and dike-segment propagation. Using the seismicity and the inferred dike dimensions, we quantify the volcanotectonic parameters associated with a magma reservoir at the anomaly‘s depth and the excess pressure needed to rupture the reservoir roof and inject dike-segments. All the results support our interpretation that the anomaly respresents the roof of the magma source reservoir that fed the 2021, 2022, and 2023 Fagradalsfjall eruptions.

### Reservoir location and rupture

**Fig. 3:**
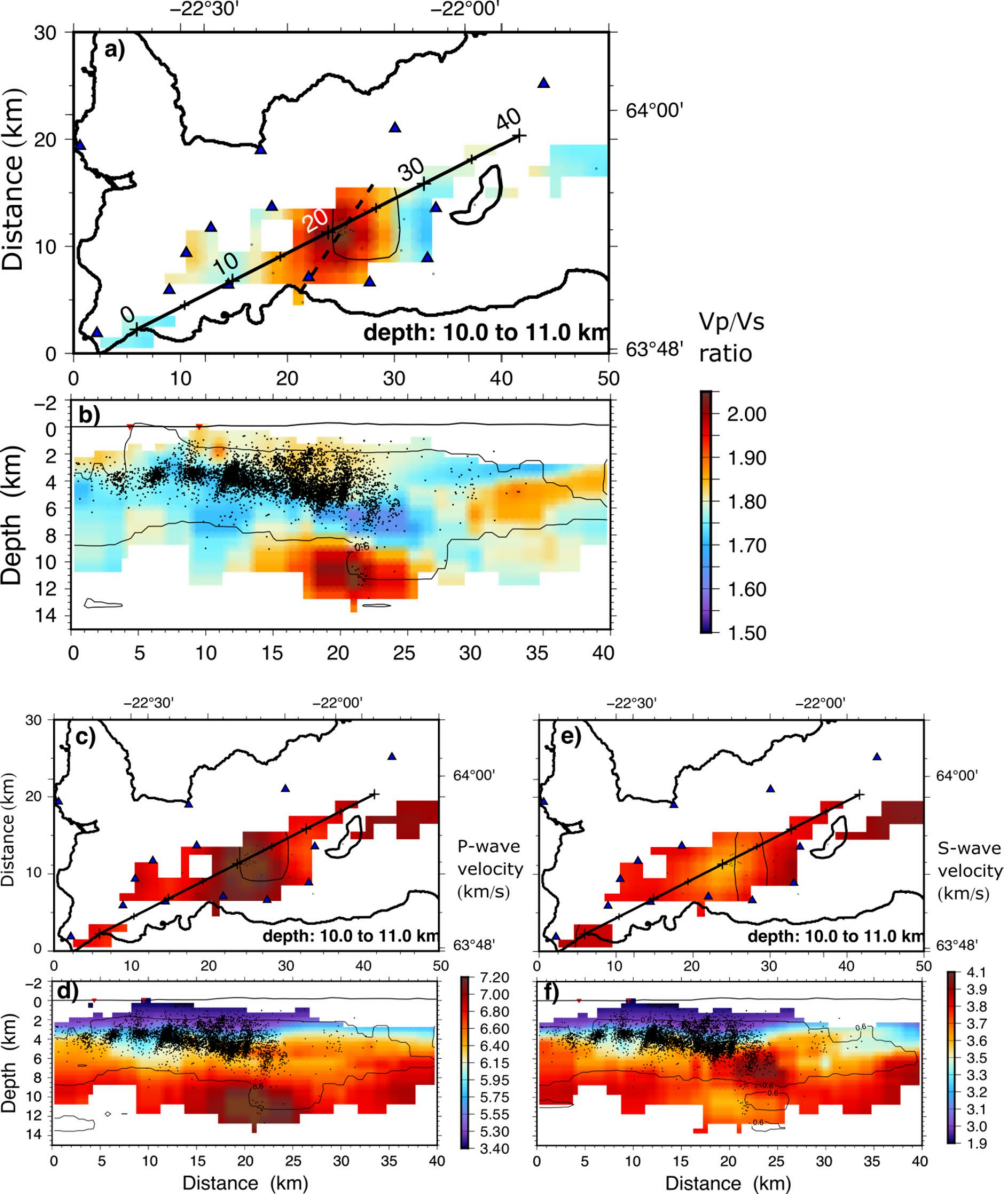
Slice at 10 to 11 km depth (**a**) and a cross section (along the line shown in the depth slice) through the final tomographic models (**b**), both showing the Vp/Vs ratio obtained by dividing the P-wave model with the S-wave model. In (**a**) only the events between 10 and 11 km depth are shown, for a map view of events from all depths see Fig. S11. Panels (**c**) and (**d**) show the P-wave velocity model and (**e**) and (**f**) the S-wave model. Seismicity within the depth slice and within 250 m on each side on the cross section is shown. Cells without rays are white. The thin line show the ‘well resolved’ regions based on the checkerboard test (Fig. S3). The stippled line in (**a**) shows the location of the cross sections in Fig. 6. The maps and cross sections were prepared with GMT version 5.4.6 (https://www.generic-mapping-tools.org) and Inkscape version 1.3.2 (https://inscape.org/).

In the present analysis we focus on earthquake data, primarily to (1) image the source region of the magma and (2) determine the rupture sites and the injection and propagation of the resulting dike through the crust. First we consider the evidence for the existence of the magma reservoir (Fig. 3), and then its rupture and dike-segment propagation - eventually to the surface (see Methods for details on the seismic data analysis and tomography).

We used abundant seismicity from December 2019 to February 2021 to produce tomographic P- and S-wave velocity images of the peninsula (Fig. 3). Due to the occurrence of seismicity at unusually large depths, a high V_p_/V_s_ anomaly was detected beneath Fagradalsfjall in the lower crust, not far from the crust-upper mantle boundary, at 11–14 km depth^14,15^. The anomaly is a robust feature in the tomographic models, which we confirmed through a number of model tests (see Methods). Explaining the cause of the high Vp/Vs anomaly as due to pressurised liquid water^15^ is not tenable, partly because of the great depth (and thus pressure) and high temperatures - far above 580 °C (the brittle-ductile transition beneath the Reykjanes Peninsula^16^ occurs at temperatures between 580 °C and 750 °C). Any available volatiles and/or meteoric fluids (unlikely to be found at these depths) must be in a compressible state, which would produce not higher but lower Vp/Vs ratios (further discussion in Methods). Since water is ruled out, we interpret the high Vp/Vs ratio anomaly at 9–12 km depth as being related to silicate melt and, therefore, indicating the location of the upper part of the source reservoir of the 2021–2023 Fagradalsfjall eruptions. 

While the reservoir may have existed for a long time^1^ prior to 2019, seismic data from earlier periods lack the depth coverage to confirm its existence. Based on the available seismic data magma was accumulating and increasing pore-fluid pressure in the upper part of the reservoir at least as early as May 2020, and possibly earlier as seismic events started occurring below 9 km depth as early as mid-December 2019. The accumulation most likely resulted in a slight magmatic excess pressure and associated stress changes in the rocks above the anomaly, that is, the roof^1^ which triggered several earthquake swarms throughout 2020 and into early 2021. From mid-December 2019 throughout 2020 there were 45 earthquakes below 9 km depth in the reservoir region. Our interpretation agrees with geochemical studies which show that the early erupted magma in the 2021 eruption was the most evolved^11^, which would normally be the comparatively low-density magma from the uppermost part of the reservoir. As the eruption progressed, the erupted magma became gradually more primitive, indicating a chemically zoned reservoir, as earlier postulated^1,5^. General considerations of magma reservoir formation and maintenance^5^ (see the section on Reservoir formation) indicate that the reservoir extends much deeper, but model tests indicate that we cannot image deeper than about 12 km.

**Fig. 4:**
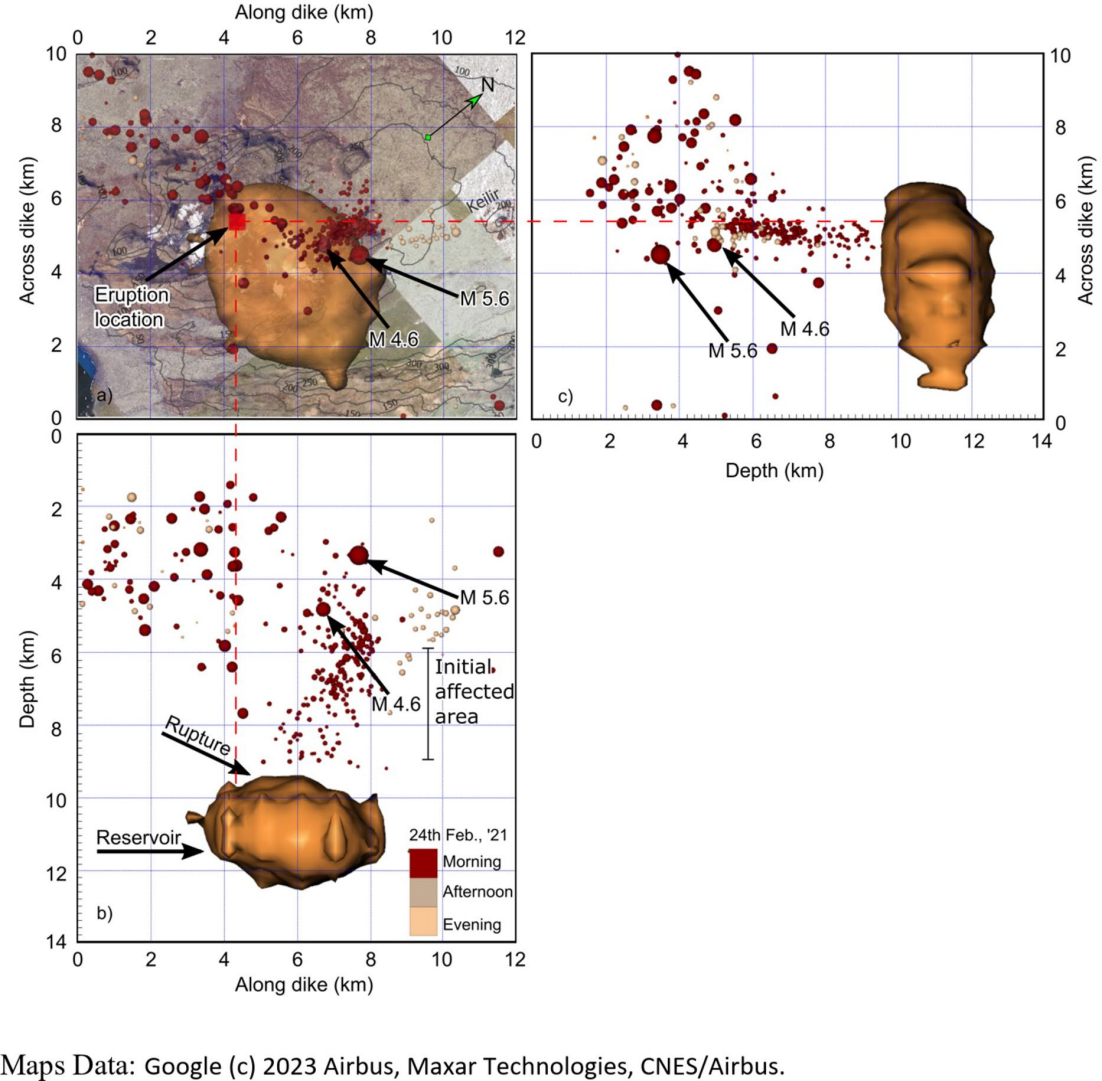
(**a**) Plan view and (**b**) vertical cross-section views of the upper part of the reservoir (brown) and the associated seismicity at the beginning of the eruptive earthquake swarm induced by the dike propagation, namely in the morning of 24 February 2021. In (**a**) the location of the 2021 eruption site at the surface is indicated (and its projection down to the reservoir (red broken line, also shown in c), as well as the M4.6 and M5.6 earthquakes (the epicentres) in relation to topography, including the landmark mountain Keilir. In (**b**) the earthquake swarm (and inferred dike) is viewed in a direction perpendicular to the inferred dike (along the normal to the dike-fracture plane). In (**c**) the earthquake swarm is viewed along the strike (direction) of the dike (along the normal to the dike-fracture opening or thickness). The dike-segment initially became arrested at a depth of ~ 2 km. The brownish volume is the high Vp/Vs anomaly (Vp/Vs > = 1.95) from the tomography in Fig. 3. This anomaly is interpreted as the shallowest part of the magma reservoir. Notice that the Vp/Vs anomaly is not resolved below the depth of 12 km^71^. The 3D images were generated by Voxler, version 4 (https://support.goldensoftware.com), the map on the plan view was downloaded from Google Earth Pro version 7.3.6.10201 (https://earth.google.com) and georeferenced in ArcGIS Pro version 3.0.36056 (https://www.esri.com), and the figure prepared in Inkscape version 1.3.1 (https://inkscape.org).

To track the path of the magma from the reservoir to the eruption site, the seismic catalogue between 24 February and 19 March 2021 was relocated in the 3D seismic models. The seismic events leading up to the main reservoir rupture and dike-segment injection on 24 February 2021 may be summarised as follows. From 2 to 3 am there were several small events in the roof of the reservoir, just above its inferred top. A larger set of aligned events just above the top indicate that rupture occurred between 3 and 5 am (Fig. 4). The following dike-induced seismicity migrated upwards to a region at 6–8 km depth, where a small swarm occurred from 6 to 10 am, eventually reaching a crustal depth of about 2 km. At 10.05 am an M4.6 event in this region was followed within a few seconds by the largest event (M5.6) in the earthquake swarm (at crustal depth of about 3 km), presumably triggered by the stress changes induced by the dike-segment (Fig. 4). This large event set off many others in the reservoir roof, including an M3.6 event. Further M2 events occurred above the reservoir later in the day, at 7–10 pm.

**Fig. 5:**
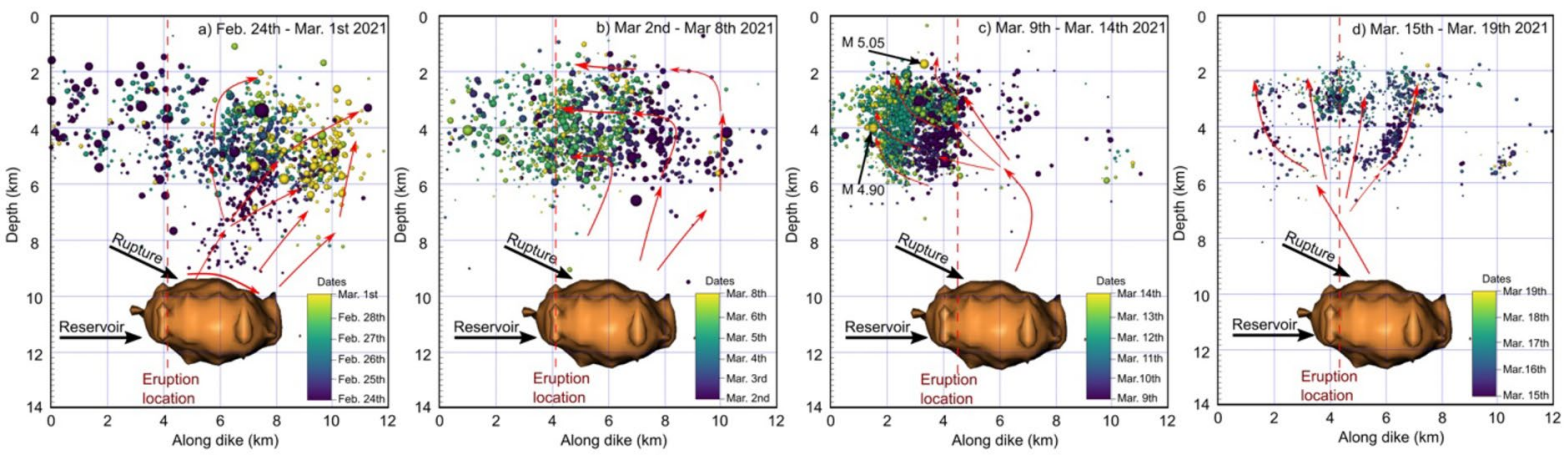
Seismicity of the eruptive swarm in 2021 and the interpreted magma flow (dike-segment propagation), indicated schematically by red arrows in four parts. The cross-section is along the dike and thus striking NE-SW. (**a**) Initial rupture and dike-segment propagation (beneath a stress barrier) to the northeast reaching ~ 2 km depth. (**b**) From the 2 March the seismicity migrated to the southwest with much seismicity inside the plate-boundary zone. The continued influx of magma caused the dike-segment to reverse its direction of propagation. (**c**) Full dike-segment propagation to the southwest after passing the plate-boundary zone on 9 March. A stable path through the boundary zone allows propagation to the southwest where the dike found a path up to shallower depths. There, two large (approximately) M5 events on 14 March were associated with changes in the propagation dynamics. (**d**) Again seismicity northeast of the boundary zone as the dike tries two possible paths/fingers to the surface. One of them leads up to the eruption on 19 March whereas the other becomes arrested close to the surface. Size shows event magnitudes. Seismicity to the west is cut off so as to focus on the eruptive swarm itself. The brownish volume is the top of the magma reservoir (Fig. 3). Top and across dike views are shown in Fig. S8. The 3D images were generated by Voxler, version 4 (https://support.goldensoftware.com) and the figure prepared in Inkscape version 1.3.1 (https://inkscape.org).

On 14 March there were two M5 events, one of which occurred about 2 km below and less than 1 km to the southwest of the eventual eruption site (Fig. 5). On 15 March three seismic swarms occurred in succession along the dike path. The first swarm was associated with the southwest part of the dike. The second swarm was to the northeast of the first and slightly southwest of the second reservoir rupture, and directly below the eventual eruption site. The third swarm occurred 2 km further northeast. Overall, some 9 km of the dike length (strike-dimension) was seismically active on 15 March, primarily at estimated crustal depths between 2 and 6 km. We interpret these seismic events as the result of new injection of magma into the dike. This interpretation is supported by the dike-segment paths inferred from induced seismicity prior to the 2022 and 2023 eruptions in Fagradalsfjall, as well as the (so far 7) eruptions at and adjacent to the nearby Sundhnukar volcanic fissure (crater row). All these eruptions have been fed by multiple dikes^5^, as is discussed below.

The emplacement of the dike-segments induced more than 30,000 earthquakes, providing a continuous seismicity from the first rupture (24 February) until the eruption began (19 March at 8.45 pm). This continuous seismicity made it possible to monitor the vertical and lateral propagation of the dike-segments.

### Reservoir formation

The flow of magma in a porous reservoir such as the one beneath Fagradalsfjall is from higher to lower potential energy^17^. This fact is represented by Darcy’s law^5^ (Eq. 1 in Methods) that applies to all fluid-filled reservoirs/aquifers that can be modelled as porous media. Studies of fossil magma chambers/reservoirs worldwide suggest that the internal flow of melt/magma is primarily porous-media flow (which applies also to dense grain-boundary-scale fracture flow) or convection in totally fluid parts of chambers^5^. Here we use Darcy’s law to explain how magma accumulated to form the Fagradalsfjall reservoir.

For the Reykjanes Peninsula (Fig. 1), regions of local minimum potential energy, and thus potential sites for magma reservoirs, are primarily due to three factors: (a) plate movements, (b) reduced lithosphere/crustal thickness, and (c) stress concentration in, and fracturing of, the lithosphere/crust. The reservoir beneath Fagradalsfjall is located at the intersection between the strike-slip (transform) plate boundary and the proposed volcanic system. The lowering of the potential energy, hence the formation of the Fagradalsfjall reservoir, is thus largely due to stress concentration and faulting as well as a likely local minimum in the crustal thickness (Fig. 2).

Estimating the volume of the Fagradalsfjall reservoir from the tomographic model is difficult, given that the resolution below 12 km depth is poor and that the reservoir must reach much greater depths. The latter follows from general considerations as to how deep-seated reservoirs are formed and maintained and, in particular, from the ratio between the volume of magma leaving a ruptured reservoir and the total volume of the reservoir^1,5^. In order to form a typical Holocene lava shield of about 1 km^3^ in a single eruption, a reservoir beneath the Reykjanes Peninsula would have to reach a thickness as great as 14–15 km^1^. Based on the Vp/Vs ratios, we may estimate the total volume of the Fagradalsfjall reservoir above 12 km depth to be between 50 and 150 km^3^. Petrological studies from the present eruptions suggest that the magma derives from storage depths of 10–17 km^11,18,19^, indicating that the volume of the Fagradalsfjall reservoir may be at least two to three times larger than in our image, but likely smaller than what could be expected for other volcanic systems on the Reykjanes Penisula^1^.

### Dike-segment initiation and propagation

**Fig. 6:**
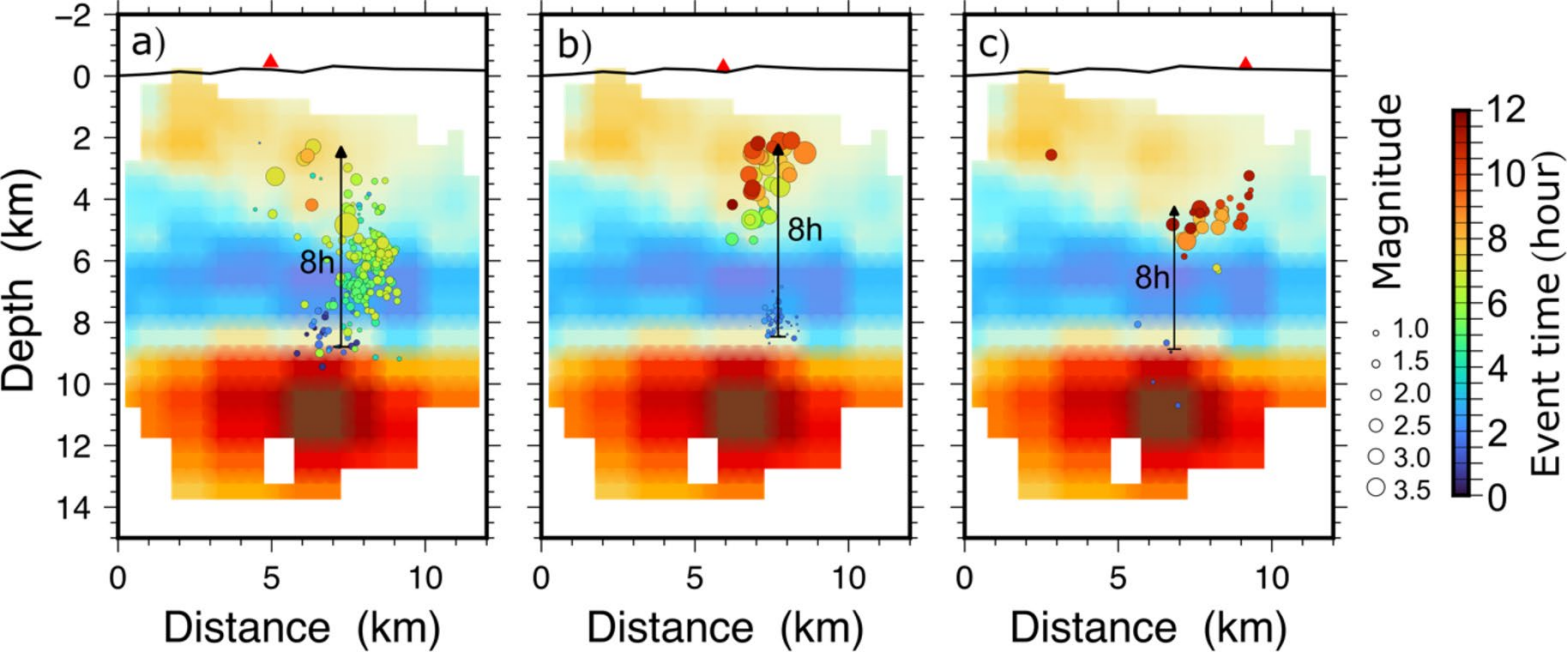
Seismicity indicating the dike propagation prior to the three Fagradalsfjall eruptions overlying the Vp/Vs ratio model. The Vp/Vs ratio colour palette and the location of the cross sections are shown in Fig. 3. (**a**) Seismicity started at about 3 am on 24 February 2021, migrating upwards and reaching about 2 km depth at noon the same day. (**b**) At about 8:30 am on 30 July 2022 seismicity started in the roof of the reservoir, reaching 2 km depth 8 h later. (**c**) On 4 July 2023 a few events within the reservoir and its roof suggest magma started migrating upwards at about 2 pm. At about 10 pm the same day seismicity had reached above 4 km depth. During the 2022 and 2023 eruptions the dike penetrated the lower crust aseismically, likely due to it following the path formed during the 2021 dike injections, but then breaking a new path at shallower levels, as is commonly seen in multiple dikes^83^. The cross sections were generated with GMT version 4.5.6 (https://www.generic-mapping-tools.org) and the figure prepared in Inkscape version 1.3.2 (https://inkscape.org).

As magma accumulated in the Fagradalsfjall reservoir, its excess pressure (*p*_*e*_ in Eq. 2 in Methods) increased (resulting in associated seismicity) until the condition for reservoir rupture and dike injection^5^ were reached. Reservoir rupture (Fig. 4) occurred on 24 February. The injected dike-segment propagated mainly vertically but became arrested at a crustal depth (depth below the surface) of about 2 km. The depth to the top of the arrested segment varied along its strike-dimension, the minimum depth being where the ‘finger-like’ feeder subsequently propagated to the surface to erupt (Figs. 4, 5 and 6). As the vertical propagation of the first segment became arrested, while it continued to receive magma, the segment spread laterally (Fig. 5), first to the northeast and then to the southwest, reaching an overall maximum strike-dimension (length) – based on earthquake data – of about 9 km.

**Fig. 7:**
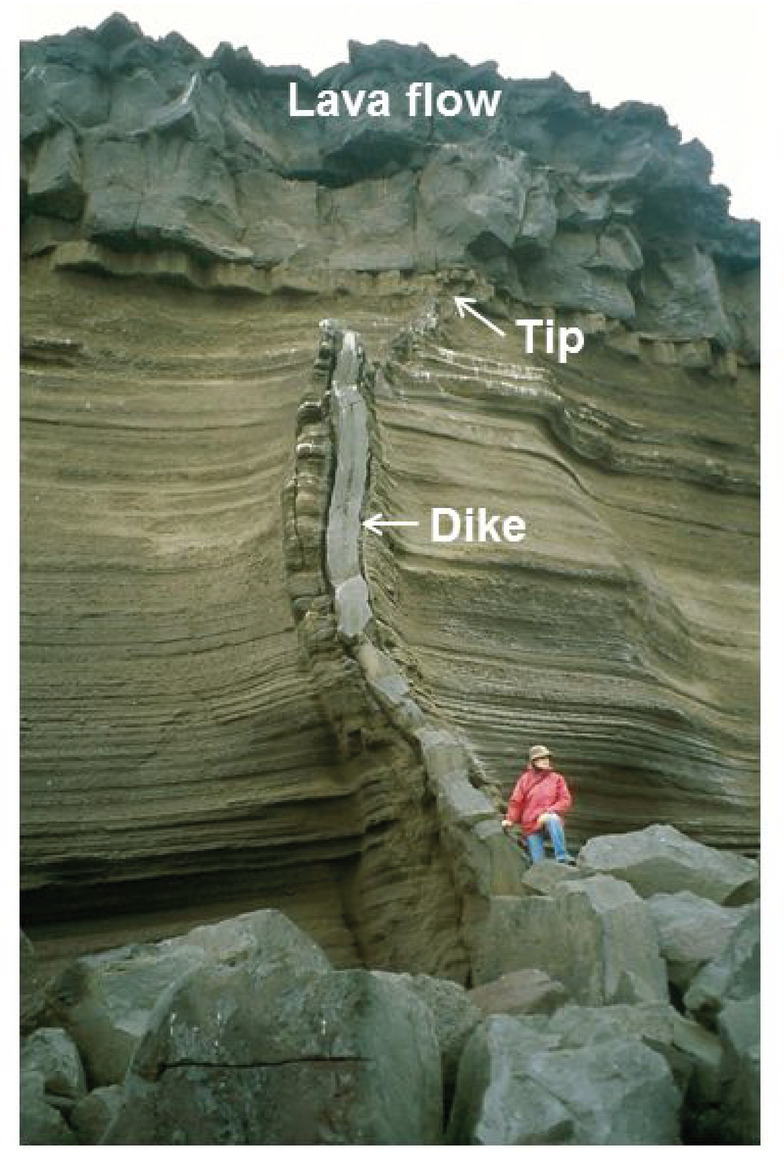
All dike-segments injected during the 2021–2023 Fagradalsfjall eruptions became arrested, initially at about 2 km depth. Dike arrest is encouraged in the crust of the Reykjanes Peninsula by the widely contrasting mechanical properties between stiff basaltic lava flows and the compliant basaltic breccia (hyaloclastite) and tuff layers. Here is an example of an arrested dike on the Reykjanes Peninsula at only 5–6 m depth below the surface about 800 years ago. The thickness of the dike is about 0.3 m at the bottom of the exposure but gradually thins to about 0.1 m at the contact between the lava flow and the welded tuff where the tip of the dike became arrested^8,85^. Thus, the basaltic dike seen here is very similar in thickness to the feeder-dike ‘fingers’ that fed the 2021–2023 eruptions. The arrested dike-segment seen here has numerous vesicles generated by gas bubbles at a very shallow depth (as expected to have happened in the Fagradalsfjall feeder ‘fingers’). One main mechanical difference, however, is that the abrupt change in Young’s modulus between the compliant brown tuff hosting the dike and the stiff Holocene lava flow at the surface, seen here, is absent at the location in Fagradalsfjall where the dike ‘fingers’ reached the surface as a feeder in the eruptions. Photo: A. Gudmundsson.

Dike arrest such as here can generally be attributed to three main mechanisms: Cook-Gordon delamination, a stress barrier, and an elastic mismatch^5^. In the Cook-Gordon mechanism, the induced dike-parallel tensile stress tends to open (delaminate) a contact of low tensile strength between layers ahead of the dike tip. On reaching the contact, the dike may then form a sill, propagate laterally, or stop altogether (no sill-formation was detected in Fagradalsfjall). In a stress barrier, the local stress field in the rock layer/unit is unfavourable to dike propagation because the maximum and intermediate compressive principal stresses σ_1_ and σ_2_ are horizontal whereas the minimum compressive principal stress σ_3_ is vertical. Such a stress field makes it normally impossible for the dike to continue its vertical propagation^5^. In an elastic mismatch mechanism it is the difference between the Young’s modulus on either side of contact in relation to the contact properties that controls dike arrest. The higher the Young’s modulus of the layer above a sub-horizontal contact, compared with to that of the layer below the contact and hosting the dike, the greater is the tendency for the dike to become arrested. Contacts between mechanically dissimilar layers are abundant in the crust of the Reykjanes Peninsula, as seen at the surface^8^ - where some arrested dikes are observed (Fig. 7) - and in deep drill holes. In the 4.6-km-deep IDDP-2 drill hole into the nearby Reykjanes Volcanic System there are many such contacts; for example, between stiff pillow lava units and compliant hyaloclastite units^20–22^.

### Rates of dike propagation

Details of the seismicity of the first 12 h of dike propagation during the 2021–2023 eruptions are shown in Fig. 6. The dike that eventually fed the 2021 eruption began its vertical propagation between 3 and 5 am on 24 February and became vertically arrested at about noon the same day (Fig. 6a). Using the estimated depth to the rupture site as 9 km and the depth where the vertical propagation of this segment initially became arrested at about 2 km, the dike segment must have propagated vertically approximately 7 km in 8 h. This implies an average rate of vertical propagation of about 0.2–0.3 m s^− 1^. Following this vertical arrest, lateral dike propagation became the main process by with the additional magma was accommodated by the dike (Fig. 5).

In 2022, magmatic pressure built up in the reservoir, indicated by increasing seismicity at depths of 8–9 km. On 30 July, after a week without seismic activity at these depths, seismicity resumed at 8:30 am, suggesting a reservoir rupture with a dike-segment injection. Magma likely reached 2 km depth by 5 pm, triggering a series of earthquakes over M4. The average rate of vertical dike-segment propagation was about 0.2 m s^− 1^ (Fig. 6b). By midnight on 31 July, a dike-segment propagated to shallower depths, with seismicity migrating 6 km to the southwest. The largest earthquake, M5.0, occurred at 2:30 am on 2 August. On 3 August, a M4.6 earthquake at 1 km depth southeast of the eruption site signaled the formation of a short fissure fed by a ‘finger-like’ dike-segment.

**Fig. 8:**
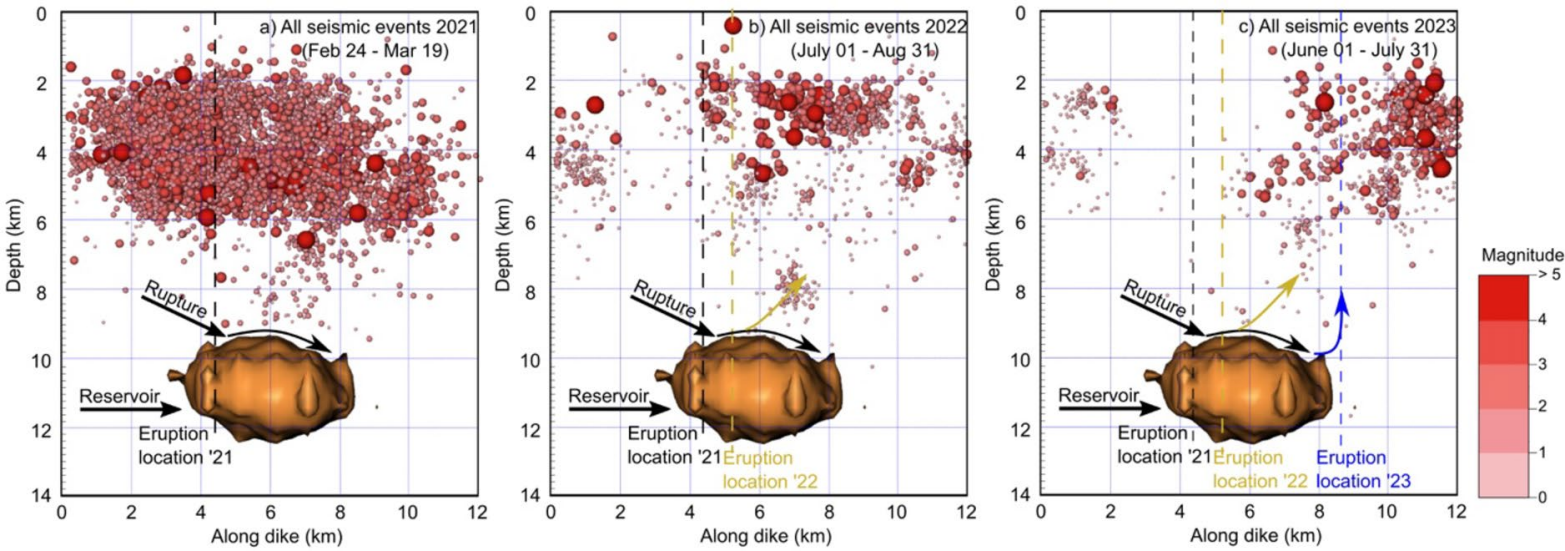
The seismicity associated with each of the 3 Fagradalsfjall eruptions. (**a**) 2021: The full length (strike-dimension) of the dike (9 km) was activated during the eruption. (**b**) 2022: Primarily the central segment of the dike emplaced in 2021 was activated during the eruption. (**c**) 2023: Primarily the northeastern segment of the dike emplaced in 2021 was activated during the eruption. The seismicity indicates that magma entered the crust from the reservoir at roughly the same location. The seismicity gives an indication where magma was injected into the multiple dike^5^. Multiple dikes are very common and are thought to make dike-fed eruptions easier and thus more likely^83^. The 3D images were generated by Voxler, version 4 (https://support.goldensoftware.com) and the figure prepared in Inkscape version 1.3.1 (https://inkscape.org).

The third eruption in Fagradalsfjall began at 4.40 pm on 10 July 2023. Prior to that eruption there were fewer deep events than before the 2022 eruption. More specifically, on 4 July several deep events were recorded in the roof of the magma reservoir, the first at 2.15 pm, and further events one hour later. At about 10 pm the same day several earthquakes occurred above the reservoir at a depth of 5–6 km. The largest was a M3.6 event. Shortly after 1 am the next day there were earthquakes at a depth of 3–4 km (Fig. 6c). This would imply a vertical propagation rate again very similar to the 0.2 m s^− 1^ propagation rates computed for the dike injections of 2021 and 2022. Earthquake activity continued during that day with two M4.5 events occurring in the evening. The earthquake activity was less in the following days, but the largest earthquake (a M5.2 event) in the region occurred on the night of the 9 July (Fig. S10). The eruption started the following day (10 July), at 4.40 pm, with the volcanic fissure opening some 5 km to the northeast of the fissures of the 2021 eruption. The 2023 eruption was thus fed by the northeast part of the multiple dike that formed in the 2021–2023 volcanotectonic events (Fig. 8). Fig. S11 shows the events plotted as depth vs. time for the three eruptions, indicating dike propagation rates ranging from 0.18 to 0.26 m s^− 1^.

### Magma overpressure and velocity

The magmatic pressure driving dike propagation at any particular crustal depth is the total fluid pressure minus the normal tectonic stress on the dike walls and is referred to as overpressure or driving pressure. It is the fluid pressure available to rupture the rock, separate the fracture walls, and advance the magma-driven fracture. For a magma-filled fracture, the overpressure *p*_*o*_ (assumed constant at a particular depth in the fracture) is given by Eq. 3 in Methods^5,23^.

Initially in the 2021 eruption (on 19 March), the length of the fissure was only about 180 m^11,12^. Subsequently, several (discontinuous and offset) segments were added to the fissure, giving it a total maximum length of about 700 m. Of these, only one main crater was active during most of the eruption. Satellite data indicate an initial fissure opening (aperture) of about 0.2 m^24^.

Using an in-situ Young’s modulus of 5 GPa and a Poisson’s ratio of 0.25^28^ a fissure length of 180 m, and an opening of 0.2 m, Eq. 3 in Methods gives the overpressure of the feeder-dike when it ruptured the surface as about 3 MPa. This low overpressure is plausible for three main reasons. (1) This value is similar to the typical in-situ tensile strength of rocks in general^5,25^ and volcanic rocks in particular^26,27^. (2) The magma is primitive and its density^11^ - about 2800 kg m^− 3^ - is greater than that of the upper part of the crust in Iceland^28^, resulting in negative buoyancy in the dike in that part of the crust and decreasing overpressure as the dike approached the surface. (3) The low overpressure and shortness of the volcanic fissure are in agreement with the low effusion rate which was mostly 4–8 m^3^ s^− 1^ at the beginning of the eruption^11–13^.

The initial effusion rate can also be used to obtain a crude estimate of the velocity of magma flow up through the volcanic fissure. The magma velocity *v* follows from the relation *v* = *Q/A*, where *Q* is the effusion rate, and *A* is the cross-sectional area of the opening of the volcanic fissure. From the initial fissure length and opening given above, we obtain the cross-sectional areas of the opening as about 36 m^2^. Using the effusion rate of 6 m^3^ s^− 1^ we get the vertical velocity of the magma in the fissure of about 0.2 m s^− 1^. Thus, the vertical velocity of the magma during the initial stages of the eruption is about the same as the average rate of propagation of the feeder-dike.

### Dike dimensions

The static Young’s modulus at 2 km depth is about 30 GPa^28^ and the common Poisson’s value for solid rocks is 0.25. At this depth, the dike overpressure is estimated at 7 MPa (Eq. 4 Methods). Using these values and a strike-dimension or length of 9 km (Fig. 5), then from Eq. 5 in Methods we get the 2021 dike thickness at this crustal depth as about 4 m. This value is very similar to common dike thicknesses in Iceland, measured in the field at crustal depths of 1–2 km below the original surface of the lava pile^5,9^. Thus, based on these considerations, and leaving out the tiny feeder-dike part (the finger), the dimensions of the main dike emplaced during the Fagradalsfjall eruption at a crustal depth of 2 km are approximately as follows: the maximum strike-dimension 9 km, the dip-dimension 7 km, and the thickness 4 m.

## Discussion

In the past few years, many papers have been published on the three Fagradalsfjall eruptions, particularly on the 2021 eruption. Some of these papers deal with surface observations such as effusion rates, lava transport^13^ and lava fountains^12^ and the petrology and geochemistry of the lavas^29^, while others focus on tectonic fractures in Fagradalsfjall^24,30^ or the tectonic stress changes prior to the 2021 eruption^31,32^. Related to the tectonic stress changes are papers on surface deformation, particularly the deformation related to the dike emplacement^33^, as well as papers on seismic swarms preceding the eruptions^34,35^. Then there are several papers on magma accumulation in the source reservoir^18,36^, its internal structure^29^, primarily from a geochemical and petrological point of view, with some papers proposing that the same reservoir feeds the eruptions in Fagradalsfjall and in the Sundhnukur crater row^19^ – the latter erupting 7 times from December 2023 to December 2024^38^.

The present work differs from and complements the above studies in several ways. First, as regards the reservoir location we focus on the analysis of tomographic data as to the reservoir location. Second, we use Darcy’s law on fluid flow in porous media to explain the reservoir formation and maintenance. Third, as regards the emplacement of the feeder-dikes, we combine earthquake and volcanotectonic data and models (including extensive datasets on dikes in Iceland as well as principles from solid/fracture mechanics) to enhance our understanding of dike propagation paths to the surface during the eruptions in Fagradalsfjall.

All the previously detected deep-seated reservoirs in Iceland are parts of double-chamber systems^2–5^ where the reservoir acts as a source for a shallow crustal magma chamber in the same volcanic system^37^. These include the double chambers of the volcanic systems of Grimsvötn^2,38,39^, Bardarbunga^40,41^, Katla^42^, Askja^3,4^, and Krafla^3,43^. Double magma chambers, and in some places triple magma chambers^44^, have also been detected beneath many volcanic systems in other parts of the world^45^. The deep-seated reservoir beneath Fagradalsfjall, however, is not associated with a shallow crustal magma chamber, and no long-lived shallow chambers are known on the Reykjanes Peninsula proper. Thus, not only is it remarkable to detect a deep-seated reservoir using seismic methods but, for Iceland, even more so because the reservoir is not a part of a double magma chamber. When such a reservoir feeds an eruption, the resulting dike must propagate largely vertically to the surface, as the current volcanotectonic episode in Fagradalsfjall has demonstrated.

We interpret the seismic results so that the main magma-reservoir roof rupture before the 2021 eruption occurred on 24 February in the shallowest part of the reservoir, at a crustal depth of about 9 km. The main rupture resulted in a dike injection and was subsequently followed by further injections using mostly the same path. The injection on 24 February became vertically arrested beneath a stress barrier at a depth initially of about 2 km. As it continued to receive magma through further magma injections, the resulting dike spread laterally (and vertically) under the barrier and reached a maximum strike-dimension (length) of about 9 km and dip-dimension (height) of about 7 km (Fig. 5). Eventually, part of the dike reached outside the stress barrier and then propagated vertically upwards as a tiny ‘finger’ (there reaching a dip-dimension of 9 km) to feed an eruption on 19 March 2021.

The seismicity associated with the reservoir ruptures suggest that the dike-injections during the eruptions of 2022 and 2023 followed mainly the northeastern part of the 2021 dike (Fig. 8) and generated a multiple dike^5^. The results from the three eruptions also demonstrate that during emplacement dikes propagate in all directions, like other fluid-driven fractures^46^. None of the arrested dike-injections during these events in 2021, 2022, and 2023 induced much surface deformation^24,47,48^, partly because of the effects of layering in suppressing dike-induced surface stresses and displacements^49–51^.

All dike-segments injected in the 2021–2023 events propagated vertically at an average rate of about 0.2 m s^− 1^, which is similar to that of many earlier estimated dike propagation rates. For example, the rate of dike-induced earthquake migration in the Krafla Volcanic System in North Iceland from 1975 to 84 was generally from 0.4 m s^− 1^ to 1.2 m s^− 1^ while the rate of lateral surface-fissure (feeder-dike segment) propagation was lower^52^ (from 0.1 m s^− 1^ to 0.4 m s^− 1^). During the dike-emplacement episode in the Manda Hararo-Dabbahu spreading centre in Africa from 2005 to 2010, the inferred rate of earthquake migration was similar^53^ (from 0.14 m s^− 1^ to 0.63 m s^− 1^) to the rates in Fagradalsfjall. Dike propagation rates in Etna, primarily inferred from surface fracturing rather than earthquake migration^54^, range from 0.02 m s^− 1^ to 0.46 m s^− 1^. Earthquakes migrated some 48 km in 13 days^7,55^ prior to the beginning of the 2014–2015 Bardabunga/Holuhraun eruption at an average rate of 0.04 m s^− 1^, much less than in Fagradalsfjall but similar to the lowest rates in Etna.

All the above propagation rates (except in Fagradalsfjall) refer primarily to lateral propagation of earthquakes and fracturing. Few comparable datasets exist on the vertical rate of earthquake migration and dike propagation. Perhaps the most relevant one is from Piton de la Fournaise in Reunion (France) where vertical dike propagation rates as high as 2 m s^− 1^ have been reported^56^, in contrast to the lateral propagation rates from 0.2 m s^− 1^ to 0.8 m s^− 1^.

When using earthquake swarms to monitor the propagation of dikes it is taken into account that many induced earthquakes occur at significant distances from the dike. Fracture-mechanics results, however, show that the greatest stress concentration, hence the most frequent earthquakes, occur in the walls and at the tips (top and bottom) of the dike-segments^23,46–51^. The same applies to fluid-driven fractures in general, whose propagation paths are routinely monitored through induced earthquake swarms^57,58^. Additionally, field studies in deeply eroded volcanic systems show clear evidence of dike-induced faulting in the host rock next to dike-segments, both in the walls of the dike-segments as well as at their tips^5,57^. Swarms of induced earthquakes can thus provide reliable information not only on the propagation paths of dikes but also their strike-dimensions and dip-dimensions and have been used to infer the propagation paths of dikes for decades^5,6,57^ through well-established methods^58–62^.

The part of the dike that reached the surface on 19 March 2021 was a tiny ‘finger’, producing a 180-m-long volcanic fissure^11^.Eventually, several other fissure segments formed, providing a cumulative fissure length of hundreds of metres. However, the segments were discontinuous, with large offsets, and not active all at the same time. In fact, during most of the 2021 eruption, only one main crater was active. The calculated magma-flow velocity and overpressure at the beginning of the eruption of the 180-m-long fissure are 0.2 m s^− 1^ and 3 MPa. This overpressure is very similar to that of the average in-situ tensile strength of volcanic rocks^27,46^. We also estimated the magmatic overpressure in the dike at the surface as about 3 MPa, and at 2 km depth as about 7 MPa. From the latter value, we calculated the thickness at 2 km depth of the main dike emplaced in 2021 as about 4 m – a value that is very similar to the average measured thickness of regional basaltic dikes at 1–2 km depth in eroded fossil volcanic systems in Iceland^9,37^.

## Methods

### Earthquake parameter data

We obtained the event parameters, as well as travel times, from the SIL catalogue. This seismic event catalogue is produced by the Icelandic Meteorological Office using the South Icelandic Lowlands (SIL) network and (due to the unrest) some stations from the Reykjanet network^63,64^ as follows^65^:

- Automated event detection and location.
- Manual quality check and phase-pick adjustment for accepted events by analysts.
- These picks are then used to obtain the final origin times and locations.

At the time there were 18 permanent seismic stations operated in the selected area, of which 5 came into operation in early 2020. We present these final locations directly using Voxler^66,67^.

### Local earthquake tomography

The seismic traveltime tomography was done with the PStomo_eq algorithm^16^. From P- and S-wave traveltimes from local earthquakes the 3D P- and S-wave velocity structure is solved for simultaneously with new hypocentral parameters. The joint velocity-hypocentral problem is decoupled with the method of Pavlis and Booker^68^. Forward traveltimes are computed with finite difference solver time3D^69,70^. The conjugate gradients solver LSQR^71^ is used for solving for the velocity structure. The process is repeated over several iterations as the problem is non-linear. After each iteration new rays are computed in the updated velocity models from the updated earthquake locations.

### Data selection for the tomography

The data were selected from the period 15 December 2019 to 27 February 2021, a few days into the swarm. A rectangular model area 90 km by 50 km was first selected based on the seismicity distribution and the location of the seismic stations. At the time there were 18 permanent seismic stations operating in the selected area, of which 5 came into operation in early 2020. For selection, events needed to have a minimum of seven picked phases (P or S), and a seismic station located within the distance of 1.5 times its focal depth to have good constraints on the hypocentral depths^72^. Some obvious outliers were also discarded. This resulted in the selection of almost 24 000 events (about 55% reduction). The reason we did not include later events for the tomography is that almost no further events occurred below 8 km depth (in the region of interest) and thus did not contribute to the imaging of the deep magma reservoir. Extending the selection further back in time did not contribute to the illumination of the area either, due to that few stations were operating in the area.

### Model parameterisation

Seismicity is not evenly distributed within the model region. In the seismically active regions, the cut-off in seismicity is generally somewhere between 4 and 6 km depth. In an attempt to adjust the model discretisation accordingly, the model discretisation was adjusted with depth. Above 6 km depth we used rectangular model blocks 0.5 km thick and 1 km in horizontal directions. Below this depth the blocks were 1 km thick and 2 km in horizontal directions. Traveltimes were computed on a uniform grid of 0.25 km to maintain sufficient accuracy.

### Model regularisation

#### Smoothness constraints

To avoid ‘wild’ velocity variations, particularly in poorly resolved model regions, the model was regularised by applying smoothing constraints. This was implemented as soft constraints in which the Laplacian of the P- and S-wave fields was minimised along with the traveltime data^73^. The weight of the Laplacian function relative to the travel time data was controlled by a weighting parameter (c.f. Equation 3 in^16^). To find a suitable value for this parameter a series of tests were performed. An ’optimal’ value would be one that results in low data variance without the velocity models having unnecessary structure. Figure S2 shows the data variance versus the squared length of the velocity models for different values of the smoother. The model length was computed as the squared model deviation from the mean velocity at every layer. We selected a smoother with the value of 10 for both models, beyond which the data fit did not improve without simultaneously increasing the model length.

#### Cross-gradients constraints for structural similarity

The P- and S-wave velocity models are only coupled to each other in the earthquake locations. It is therefore common to employ a further constraint to limit the ratio between the models (Vp/Vs ratio damping). Here we applied a structural constraint between the P – and S-wave velocity models, by requiring that the cross product of the gradients in the respective models is everywhere zero. The resulting models are thus structurally similar^74,75^, without any constraints having been placed directly on the Vp/Vs ratios. It is a reasonable assumption that variation in state variables and composition affect both the P- and S-wave velocities, though it is well known that rigidity and compressibility are differently affected. The cross-gradient constraints do not force the P-and S-wave velocity models to be similarly affected (as would a Vp/Vs ratio damping). The assumption is rather that variations in the P-and S-wave velocity models occur at the same locations. A change in one model may thus be opposite to the change in another model. Synthetic tests show that this results in more realistic Vp/Vs ratios than if e.g. damping towards a pre-defined Vp/Vs ratio is implemented^75^.

### Final model

The final velocity models are discussed in the main text. A Vp/Vs ratio high is shown between 9 and 12 km depth at about 20 km along the cross section beneath Fagradalsfjall (Fig. 3) that is interpreted to represent the upper part of a magma reservoir located in the lower crust close to the crust-upper mantle boundary and feeding the 2021–2023 Fagradalsfjall eruptions.

### Model appraisal

#### Checkerboard reconstructions

Demonstrating synthetic checkerboard reconstruction tests is a common way to show which parts of a seismic model is robust. In those, checkers of alternating velocity perturbations are superimposed on a starting model and synthetic travel times are computed. Sometimes noise is added to this data, which is then inverted. Regions in which the checkers are well reconstructed are then deemed reliable in the real models. This may provide a general overview of which parts of the models are well resolved. To make the test ‘difficult’ and demonstrate that the cross-gradients constraints do not damp the Vp/Vs ratios per se, the P- and S-wave velocity checkers have reversed polarities. Below 6 km depth the checkers are larger, a consequence of the inversion cells being larger below this depth. Figure S3 shows the result of Vp/Vs ratio of the ‘true’ (synthetic) and the reconstructed models in the right and left panels, respectively. The Vp/Vs ratio is well reconstructed between 1 and 7 km depth, in general where seismicity occurs. In the region of the high Vp/Vs ratio anomaly in the depth range of 9 to 12 km depth we note that the checker is reconstructed, though not to its full strength. The coefficient of correlation between the reconstructed and original checkers is computed and the outline of the 0.6 isoline is shown in Figs 3 and S3 to outline were the checkers are fairly well reconstructed.

#### Hypothesis tests

To make targeted tests for certain features in a model, a specific anomaly may be removed from the final model. A few more iterations are then performed with the real data in order to investigate if the anomaly is required by the data, i.e. if the removed features are ‘put back’ into the models by the data. In Fig. S4 we have replaced the high Vp/Vs anomaly below 9 km depth with ‘normal’ velocities (panels a and c), resulting in a worse data fit. The result after a final few iterations demonstrate that the anomaly is put back by the data, returning to the previous data fit (panels b and d).

#### Characteristic model test

Whereas a checkerboard test may provide an overview of which parts of a model is well resolved, other synthetic models may be investigated to answer specific questions about the model. Fig. S5 is a synthetic model showing a region of high Vp/Vs ratios, about 6 km across and extending to great depth (right panel). The high Vp/Vs body also features a core of even higher Vp/Vs ratios (2.04). In the synthetic model a low Vp/Vs ratio layer resides above the high Vp/Vs ratio region. The model has some resemblance with the final model. The reconstruction shows that the region of high Vp/Vs ratios is clearly observed. It is, however, broader than the true model, and the core is not recovered. The depth extent of the Vp/Vs anomaly is also not possible to determine. The reconstruction also shows that the low Vp/Vs ratio layer is well reconstructed in the center of the model, but smeared out towards the south west in the model. Fig. S6 shows a much broader high Vp/Vs ratio anomaly (14 km across). In the reconstruction this model is also smeared out, but less so than the smaller anomaly in Fig. S5.

#### Lessons learned from the model tests

The hypothesis test shows that high Vp/Vs ratio anomaly beneath Fagradalsfjall at depths between 9 and 12 km (Fig. 3) is required by the data. When the anomaly is replaced by ‘normal’ velocities in the hypothesis test, the inversion puts it back into the model (Fig. S4). We also tested many different starting models (not shown), and the feature is persistent in the models regardless of starting models. The checkerboard reconstruction test (Fig. S3) shows that in the centre of the model the checkers are well reconstructed down to 8 km depth. Below this depth, the checkers are smeared out. Two characteristic model tests (Figs. S5 and S6) show that the dimensions of the high Vp/Vs ratio anomaly are difficult to determine. A 6 km wide anomaly appears wider than it is. Testing for a much wider feature (14 km) shows that even though it is smeared out, the anomaly would certainly be wider than in our results. From this we infer that the top part of the magma reservoir is likely 10 km or smaller in the horizontal direction. These tests indicate that the shallowest part of the reservoir at 9 km depth is robustly reconstructed, but also that it is impossible for us to determine its depth extent.

### Relocated seismicity

The entire seismic catalogue between 24 February and 15 March 2021, and similarly for the 2022 and 2023 eruptions, was relocated in the final velocity models. The location errors (1 SD) are shown in Fig. S7. The results indicate that the events are most accurately located in the east-west direction, probably due to the east-west elongation of the seismic network. The depth is also the parameter with the largest error. Some 90% of the relocated events have smaller depth uncertainty than 0.6 km, and 60% of the events have an uncertainty in depth of 0.3 km or less.

Further support for the high Vp/Vs anomaly indicating a magma reservoir.

Though Vp/Vs interpretation is non-unique, as is common in geophysics, the following observations support our interpretation as to the high Vp/Vs anomaly coinciding with the top of the source magma reservoir at about 9 km depth below the eventual eruption site. (1) The location of the anomaly below the eruption site. (2) The events, particularly the earthquake swarms and their migration, leading up to the eruption. (3) The eruption itself and particularly the location of the volcanic fissure. (4) The long-period seismicity coinciding with the anomaly. (5) The geochemical and petrological results indicating that the primitive magma came from a deep-seated reservoir and not from a shallow magma chamber^11,18,19,29,36^.

That the high Vp/Vs would be due to pressurised liquid water^76^ is not tenable for the present anomaly, partly because of the great depth (and thus pressure) and high temperatures - far above 580 °C (the brittle-ductile transition beneath the Reykjanes Peninsula^16^ occurs at temperatures between 580 °C and 750 °C). Any available volatiles and/or meteoric fluids (unlikely to be found at these depths), must be in a compressible state, which would produce not higher but lower Vp/Vs ratios. The propagation of seismicity, which starts at the top of the high Vp/Vs anomaly, is also a very specific signal favouring our interpretation of the anomaly as the top of a magma reservoir.

The location of the anomaly also coincides with where one would expect the source reservoir of the eruption. This follows, first, because the path of a regional dike, such as the present one, is normally close to vertical^5,57^. Second, as indicated by Eq. (1) and discussed earlier, a reservoir would be expected to be directly below the boundary zone (Fig. 1b), where the crust is comparatively thin and highly fractured (Fig. 2), namely at the site of a local potential energy minimum. Third, the roof of the reservoir should be located where the dike-propagation induced seismicity begins, as is the case here. Fourth, as the source region deepens with time, we would expect the seismicity to deepen as well (as is seen in syn-eruptive seismicity). The deep long-period (DLP) seismicity at the location of the reservoir^77^ could also be expected to occur inside a reservoir preceding and during an eruption. The reservoir is a poroelastic body and thus only partially molten – most of the reservoir is solid^5^. Several mechanisms have been proposed for earthquakes inside magma reservoirs^58,78–80^, but the most likely one is increase in pore-fluid pressure due to melt/magma accumulation. Pore-fluid pressure increase triggers earthquakes on existing fractures, as well as on new fractures, as is well known from rock-physics experiments for developing enhanced geothermal (EGS) and hydrocarbon reservoirs^58,67,81,82^. Melt migration and pore-fluid pressure changes follow from the anomaly being the top of the source reservoir, the roof rupture, and dike-segment injections. Thus, in our interpretation, DLP events at the observed depth (10–12 km) are exactly as expected.

Most of the petrological and geochemical results of Halldorson et al.^18^ (their Fig. 4) and Troll et al.^19^ agree with the top of the reservoir being at a depth of about 9 km. The fountain tephra results, which Halldorson et al.^18^ base most of their interpretation on, suggest a greater depth for the origin of the magma, but that magma was not issued from the fissure until 38 days into the eruption (see their Fig. 3a). By that time, some 20 million m^3^ of material had already erupted^13^. The source depth of 15 km depth for magma erupted after 38 days of eruption^11^ is in perfect harmony with our reservoir model. Although there were not enough seismic signals to image the deeper parts of the reservoir, volcanotectonic and petrological considerations indicate that the reservoirs beneath the Reykjanes Peninsula, including that of Fagradalsfjall, are many kilometres deep^1,5,19^ (Fig. 2). As the eruption progresses, the magma is drawn from deeper parts of the reservoir, in accordance with Eq. (1) below. More specifically, Halldorsson et al.^18^ (see also Bindeman et al.^11^) show that the magma source deepened during the 2021 eruption: the magma erupted early in the eruption was more depleted and came from a source at lower pressure than later in the eruption, when the magma was more enriched and from a greater pressure. Thus a stratified reservoir is a likely source – as has been suggested for reservoirs in Iceland in general and for those beneath the Reykjanes Peninsula in particular^1,5^. In such a reservoir, the depleted magma in the top part of the reservoir would be the first to erupt, whereas the enriched would erupt as the eruption progressed – exactly as observed in the present eruption. We conclude that, although the resolution at depth is not good for small features, the top part of the reservoir is properly resolved.

### Reservoir modelling

#### Darcy’s law

Darcy’s law for flow in porous media may be expressed as follows^5,17^:

1 $$\vec{q} = \frac{Q}{A} = - \frac{{k\rho _{m} g}}{{\mu _{m} }}\nabla \emptyset$$

Here the vector $\vec{q}$ is the discharge or Darcy velocity and denotes the volumetric flow rate *Q* per unit area *A* normal to the direction of flow, *k* denotes the intrinsic permeability, ρ_m_ the density of the magma or melt, g the acceleration due to gravity, and μm the dynamic (absolute) viscosity of the magma/melt. The symbol $\emptyset$ denotes the potential energy or total head of the magma/melt, and ∇∅ is thus the gradient of the potential energy. The negative sign is to indicate that magma/melt migration is driven from regions of higher potential energy (head) to regions of lower potential energy. Thus, magma/melt in a partially molten upper mantle is driven to regions of local minimum potential energy. While part of the magma/melt flow in the reservoir may be through fractures, in which case the cubic law would apply, the fractures are likely to be mostly of grain-size dimensions and their fluid transport may be approximated through equivalent porous flow^5,18^.

#### Conditions for reservoir rupture

The conditions for magma-reservoir rupture is as follows^5^:

2 $$\:{p}_{t}={p}_{l}+{p}_{e}={\sigma\:}_{3}+{T}_{0}$$

Here $p_{t} = p_{l} + p_{e} ~$ denotes the total magmatic pressure in the reservoir, p_l_ the lithostatic stress at the site of the rupture of the roof of the reservoir, p_e_ the reservoir excess magmatic pressure, that is, the pressure in excess of σ_3_, the minimum compressive (maximum tensile) principal stress, and T_0_ the local in situ tensile strength of the roof at the rupture site. Eq. (2) implies that when the total fluid pressure in the reservoir reaches the minimum principal compressive stress plus the in-situ tensile strength, the chamber roof ruptures and a magma-filled fracture, here a dike-segment, is injected.

### Magmatic overpressure and dike dimensions

#### Magmatic overpressure

For a magma-filled fracture, the overpressure *p*_*o*_ (assumed constant at a given depth in the fracture) is obtained from the following Eqs. 5 ref^24^:

3 $$p_{0} = \frac{{\Delta u_{I} E}}{{2L\left( {1 - v^{2} } \right)}}$$

where Δu_I_ is the opening of the dike fracture,* E* is Young’s modulus and* ν* is Poisson’s ratio of the host rock, and* L* is the strike-dimension or length of the dike fracture.

When a dike-segment begins to propagate up into the reservoir roof towards the surface, the magmatic overpressure *p*_*o*_ in the dike-segment (magma-filled fracture) can be estimated thus^5^

4 $$\:{p}_{0}={p}_{e}+\left({\rho\:}_{r}-{\rho\:}_{m}\right)gh+{\sigma\:}_{d}$$

where *p*_*e*_ is the excess magmatic pressure in the reservoir at the time of roof rupture (equal to the in-situ tensile strength of the roof rock), *ρ*_*r*_ is the average host-rock density, *ρ*_*m*_ is the average fluid (magma) density, *g* is the acceleration due to gravity, *h* is the dip-dimension of the dike (the height of the dike-segment above its source), and *σ*_*d*_ is the differential stress (the difference between the maximum and the minimum principal stress) in the host rock at the location where the dike overpressure is of interest. In Eq. (4) the term $\:\left({\rho\:}_{r}-{\rho\:}_{m}\right)gh$ represents the buoyancy.

We focus on the dike thickness at a crustal depth of 2 km where much of each of the dike-injections of 2021, 2022, and 2023 became arrested (Figs. 5, 6 and 8, S8 and S9). Here we consider the 2021 injection, when much of the multiple dike was emplaced. If we use typical generalised crustal density values for the units that constitute the crust of Iceland^28^, the reservoir depth of 9 km, the excess pressure/ in-situ tensile strength of the roof as 3 MPa^5^, the above magma density of 2800 kg m^− 3^, and *σ*_*d*_ as 1 MPa^5^, Eq. (4) gives the dike overpressure at 2 km depth as about 7 MPa. To obtain the dike thickness, we rewrite Eq. (3) and solve for the dike opening or thickness thus:

5 $$\:{\Delta\:}{u}_{I}=\frac{2{p}_{0}\left(1-{v}^{2}\right)L}{E}\:\:\:$$

which yields about 4 m thickness at 2 km depth.

#### Comparison with other dike-dimension and dike-overpressure estimates

Other estimates of the dimensions of the dike are generally similar to our results. Thus, the strike-dimension is estimated at about 9 km^32,48^. A dip-dimension of 8 km has also been estimated, but in some cases as the depth from the volcanic fissure at the surface to the base of the dike^48^. In our estimate, however, the 7 km dip-dimension is that of the main (arrested part of the) dike, from a crustal depth of 2 km to the depth of 9 km (the top of the magma reservoir). The maximum thickness (dike-fracture opening) estimate using the model of elastic dislocations in an elastic half-space^48^ (that is, a homogeneous and isotropic crustal segment, so with no layering) yields about 3 m. This is not greatly different from our result of 4 m which, though, is based on an entirely different approach using layering and principles from fluid mechanics and fracture mechanics.

Elastic-dislocation models are not suitable for explaining dike-propagation paths, however. For these, appropriate principles of fluid and solid (including fracture) mechanics are needed. The mechanical details as to how dikes select their propagation paths are given elsewhere^5,57^, but in the present paper we infer the paths of the main dike-segments from 2021, 2022, and 2023, from rupture and segment injection in the roof of the reservoir, to arrest of the main part of the dike, and finally to the eventual eruption and volcanic fissure formation of a tiny part of the main dike (Fig. 5).

One main parameter for dike propagation is the magmatic driving pressure or overpressure. This is the pressure that ruptures the crust, drives the dike propagation, and contributes largely to the eventual dike dimensions. One model of the 2021 eruption has a 13 km tall vertical pipe, with a lateral cross-sectional area of a few square metres, transporting magma from a depth of 19 km in the mantle to the base of the dike^48^. We know of no plausible mechanisms by which a pipe of such dimensions could be generated, particularly given the short time window for its formation. In this model a constant density difference of 300 kg m^− 3^ is assumed between the host rock and the magma up to the base of the dike^48^. For a 13 km tall pipe, the overpressure would then be at least 38 MPa at the base of the dike. Since the model also assumes no density difference between the rock and magma in the dike itself, the 38 MPa overpressure should be largely maintained inside the dike.

With an overpressure of 38 MPa then, from Eq. (5) and using the same elastic constants as above, the thickness of the dike would be about 14 m for a dike dip-dimension of 6 km and about 19 m for a dip-dimension of 8 km - thus including the feeder-dike part. (The authors use a dike dip-dimension of 8 km in part of their paper and 6 km in other parts.) These openings or dike thicknesses are 5–6 times larger than the maximum thickness of 3 m that the authors estimate using their dislocation model. If the same dike overpressure would be assumed to extend to the surface (8 km dip-dimension), then, from Eq. (5), the opening of the 180-m-long volcanic fissure at the beginning of the eruption would have been about 2.6 m, or more than 12-times the estimated opening^24^.

Using Eq. (4) for a layered crust, we estimate the maximum magmatic overpressure in the dike at 2 km depth as about 7 MPa. Using this overpressure, the maximum dike thickness at 2 km depth becomes about 4 m. Similarly, from Eq. (5) and the length/aperture ratio of the volcanic fissure, we obtain the overpressure of the feeder-dike at the surface as about 3 MPa.

The results also indicate that all the dike-injections during the events of 2021, 2022, and 2023 used largely the same paths. More specifically, the injections of 2022 and 2023 used parts of the already established dike from the main 2021 injection as their paths, resulting in a multiple dike. Such dikes are very common and are thought to make eruptions mechanically easier and thus more likely^83^.

## Electronic supplementary material

Below is the link to the electronic supplementary material.

Supplementary Material 1

## Data Availability

The datasets generated during and/or analysed during the current study are available from the corresponding author on reasonable request.
