# Supplementary material for: Tomographic and volcanotectonic control on the 2021–2023 Fagradalsfjall eruptions, Iceland
Source: Sci Rep. 2025 May 12;15:16455. doi: 10.1038/s41598-025-95169-6 (PMC12069532; doi:10.1038/s41598-025-95169-6)
Supplement: Supplementary file 1 — Supplementary Material 1 [file 41598_2025_95169_MOESM1_ESM.docx]

**Tomographic and volcanotectonic control on the 2021-2023 Fagradalsfjall eruptions, Iceland**

Alex Hobé^1^, Mohsen Bazargan^1^, Burcu Selek^1^, Ari Tryggvason^1,*^, Emmanuel Alofe^1^, Agust Gudmundsson^2^

^1.^ Department of Earth Sciences, Uppsala University, Villavägen 16, 752 36, Uppsala, Sweden

^2.^ Department of Earth Sciences, Queen's Building, Royal Holloway University of London, Egham TW20 0EX, UK

* Corresponding author: (Ari Tryggvason [ari.tryggvason@geo.uu.se](mailto:ari.tryggvason@geo.uu.se))

| **Supplementary material:**  Figure S1. Wadati diagram of the traveltimes used.  Figure S2. Data fit vs model length for different smoothing weight.  Figure S3. Checkerboard reconstruction test.  Figure S4. Hypothesis test.  Figure S5. Reconstruction test 1 (small anomaly).  Figure S6. Reconstruction test 2 (large anomaly).  Figure S7. Hypocenter parameter uncertainties.  Figure S8. Seismicity of the eruptive swarm in 2021– all panels.  Figure S9. Seismicity of the eruptive swarm in 2022.  Figure S10. Seismicity of the eruptive swarm in 2023.  Figure S11. Depth vs time for the events of the three dike injections. |  |
| --- | --- |

**Figure S1.** Wadati diagram of the selected traveltimes. The thin red line in the center of the cluster of traveltimes is 1.8, and the two outer lines are ±7% (1.67 – 1.92) showing that from about 10 s (P-wave time) the average variation stays within these bounds. At shorter times variation may exceed this variation, indicating that locally the Vp/Vs ratio deviates significantly from a value of 1.8 and that large Vp/Vs anomalies may exist in the 3D velocity structure of the region. The figure was prepared with GMT version 5.4.6 (<https://www.generic-mapping-tools.org>).

**
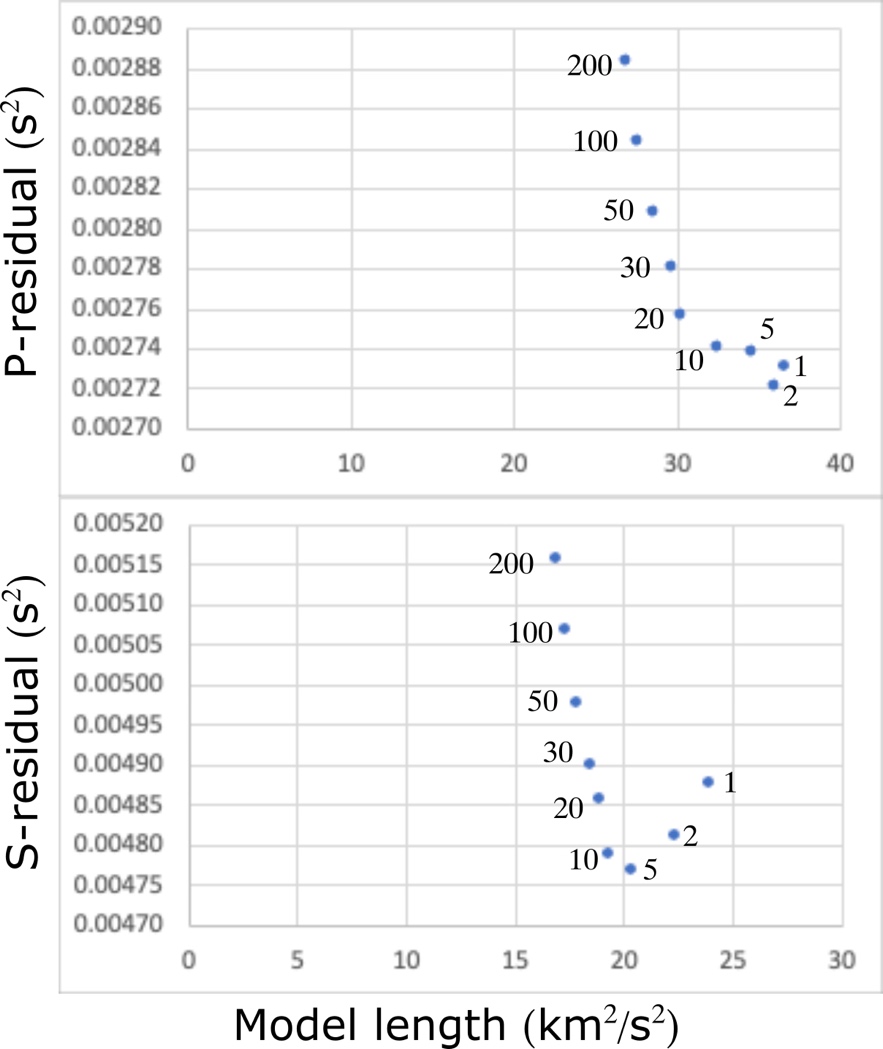
**

**Figure S2.** Weight on the smoothing constraints were selected based on a series of tests with different values. A value of 10 gave the least data residual variance without unnecessary model length. Smaller values gave more model artifacts, and larger smoothing caused a larger data misfit. The figure was prepared with Microsoft Excel version 16.78 (<https://www.microsoft.com/>).


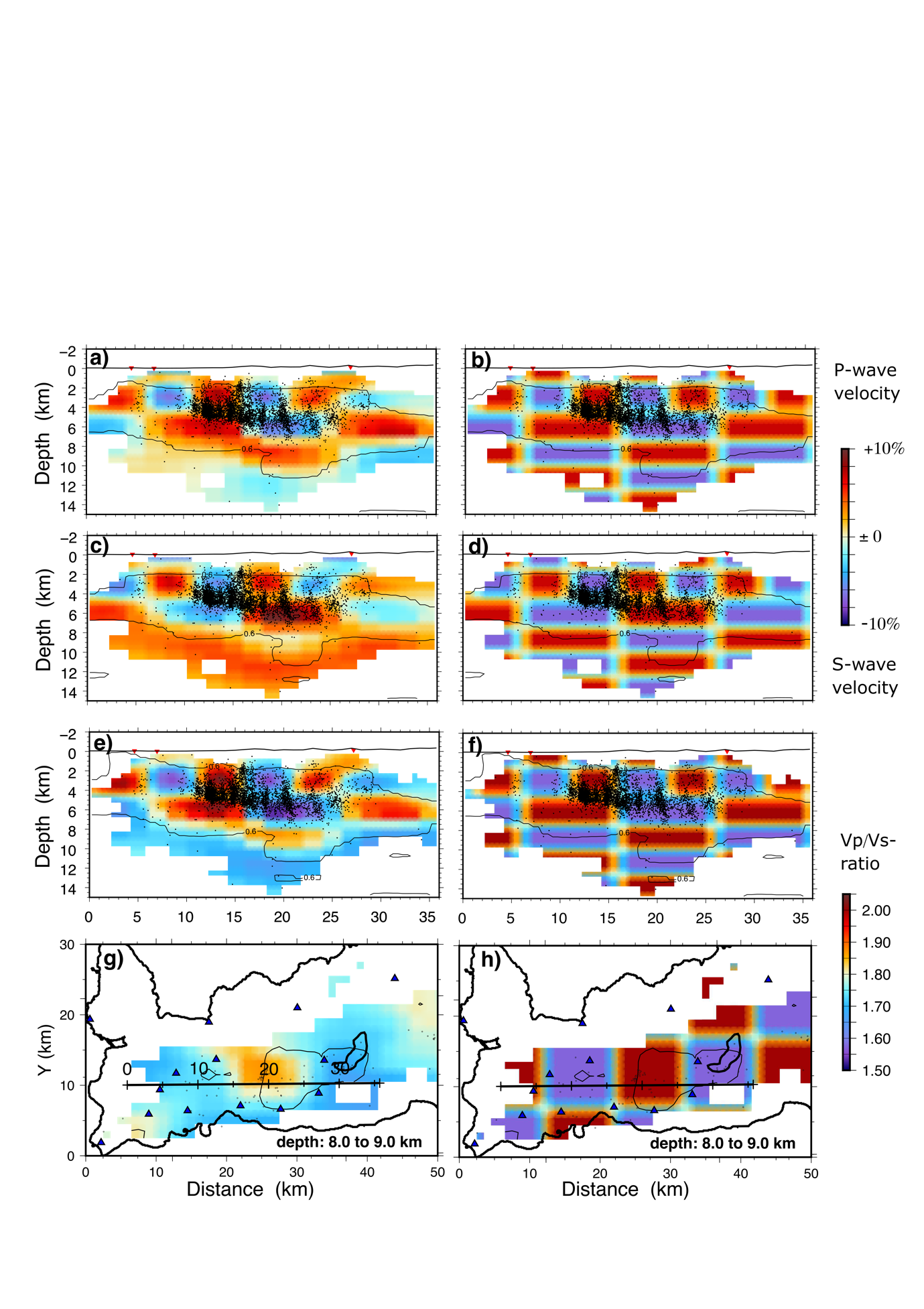


**Figure S3.** Result of the checkerboard reconstructions. A maximum of 7% velocity perturbations to the starting models were added to (and subtracted from) the P- and S-wave models. (a) and (b) show the reconstructed and true S-wave models, respectively. (c) and (d) show the reconstructed and true P-wave models and (e) and (f) show the Vp/Vs ratio. (g) and (h) show a map view at 8-9 km depth for the Vp/Vs ratio. The solid black line indicates the location of the cross sections in the panels above. Cells without rays are white. The thin contour line outlines the regions where the cross correlation between the constructed and true checkers are above 0.6, thus where the checkers are fairly well reconstructed. The map views and cross sections were prepared with GMT version 5.4.6 (<https://www.generic-mapping-tools.org>) and Inkscape version 1.3.2 (<https://inkscape.org/>).

**
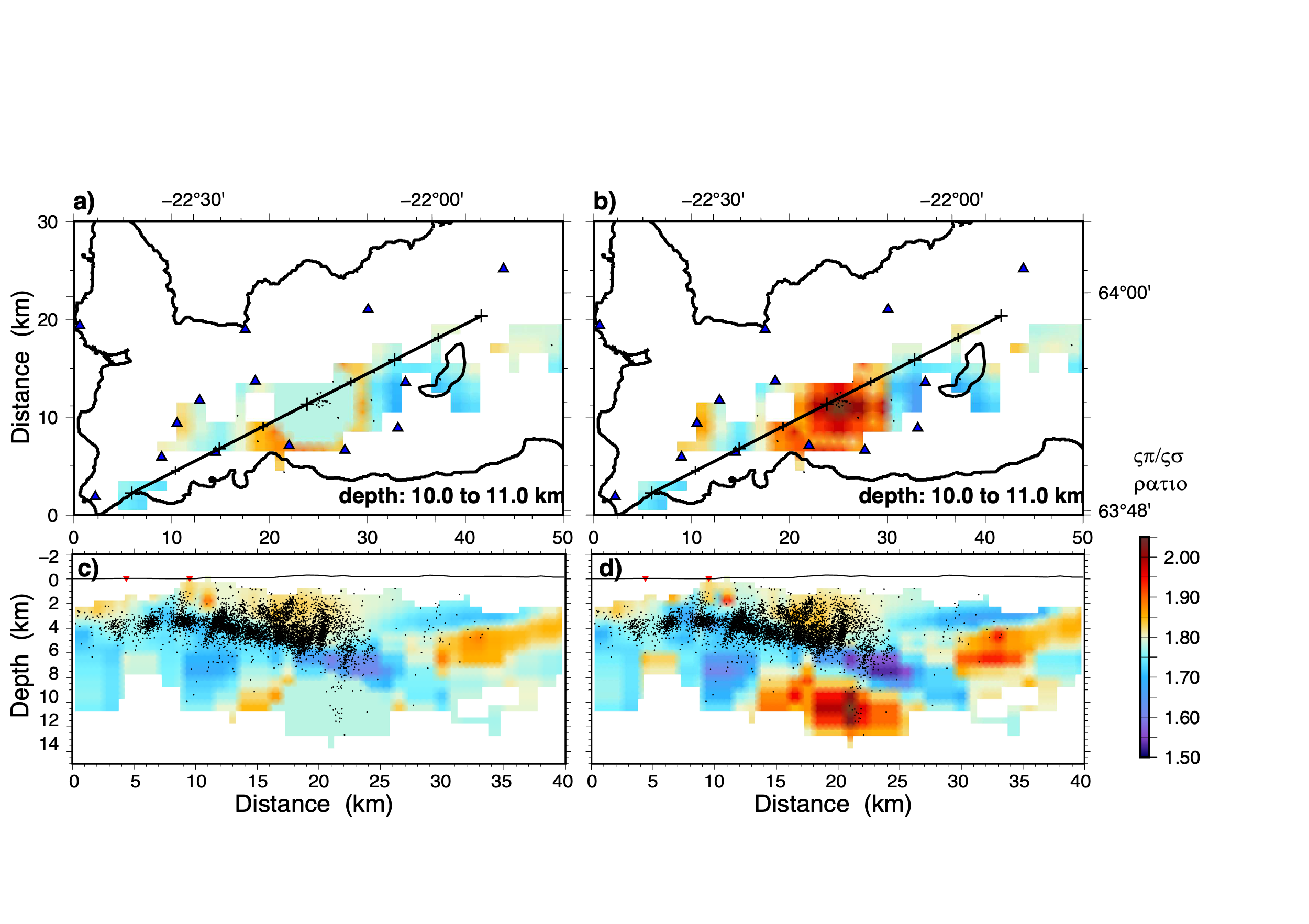
**

**Figure S4.** Result of a hypothesis test. (a) The high Vp/Vs ratio is taken out of the model (by replacing the velocities with the starting model. (b) shows the result after a few more iterations. The result indicates that the high Vp/Vs ratio is put back into the model. (c) and (d) are cross sections through the modified and reconstructed models, respectively. The result shows that high Vp/Vs ratio at 9-12 is required by the data. The maps and cross sections were prepared with GMT version 5.4.6 (<https://www.generic-mapping-tools.org>) and Inkscape version 1.3.2 (<https://inkscape.org/>).


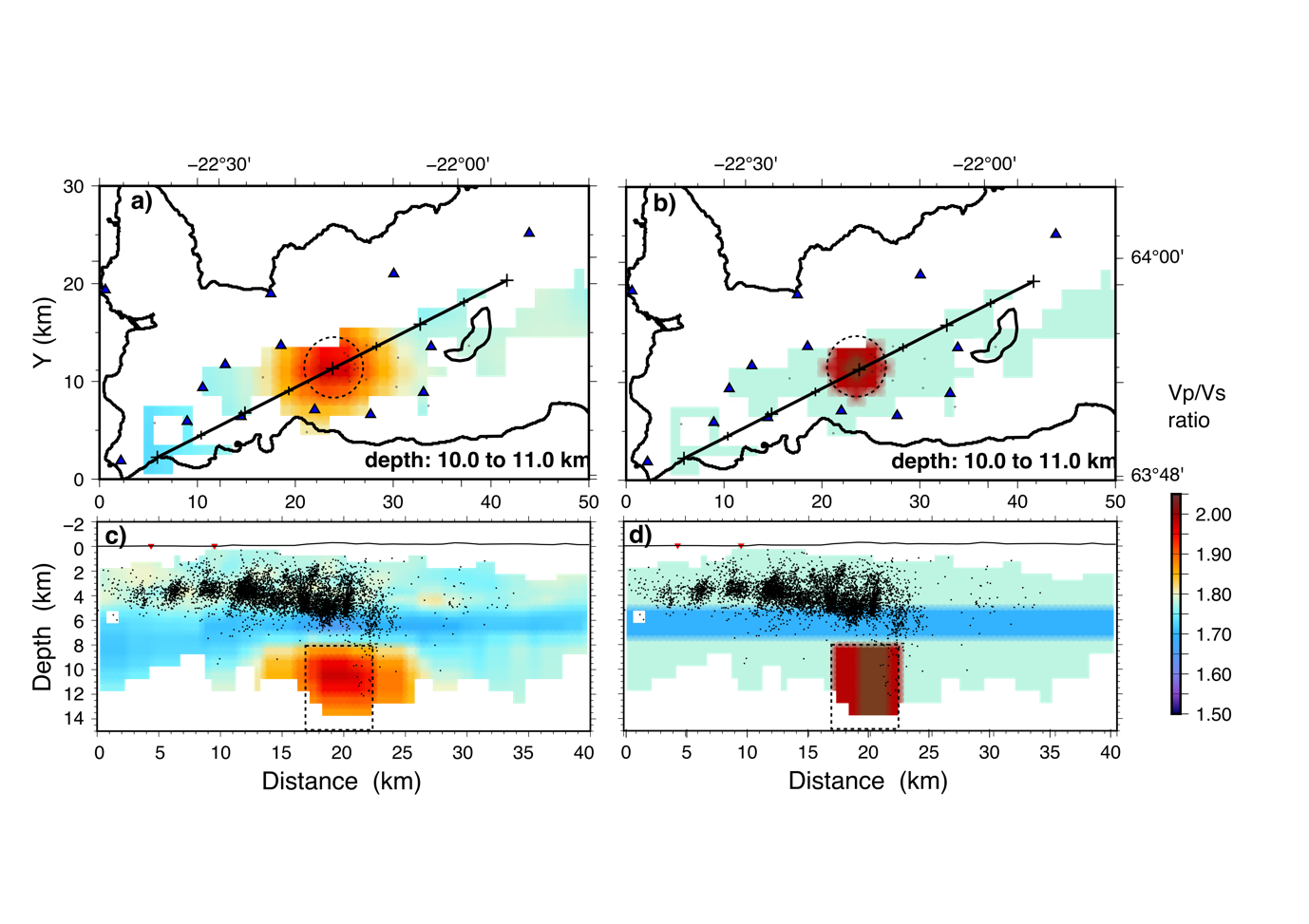


**Figure S5.** Reconstruction test with a high Vp/Vs ratio anomaly beneath Fagradalsfjall, starting at 8 km depth and extending to the bottom of the model. Vp/Vs ratio as high as 2.04 are present in its core. The anomaly is 6 km across. Above it is a layer of low Vp/Vs ratios. (a) and (b) are map views of the reconstructed and true models, respectively. (c) and (d) show cross sections through the models. The high Vp/Vs ratio anomaly is clearly observed, though it appears wider than in the true model. The depth extent of the anomaly cannot be determined. The low Vp/Vs ratio layer is fairly well reconstructed in the center of the model, but smeared out in the southwestern corner of the model. The maps and cross sections were prepared with GMT version 5.4.6 (<https://www.generic-mapping-tools.org>) and Inkscape version 1.3.2 (<https://inkscape.org/>).


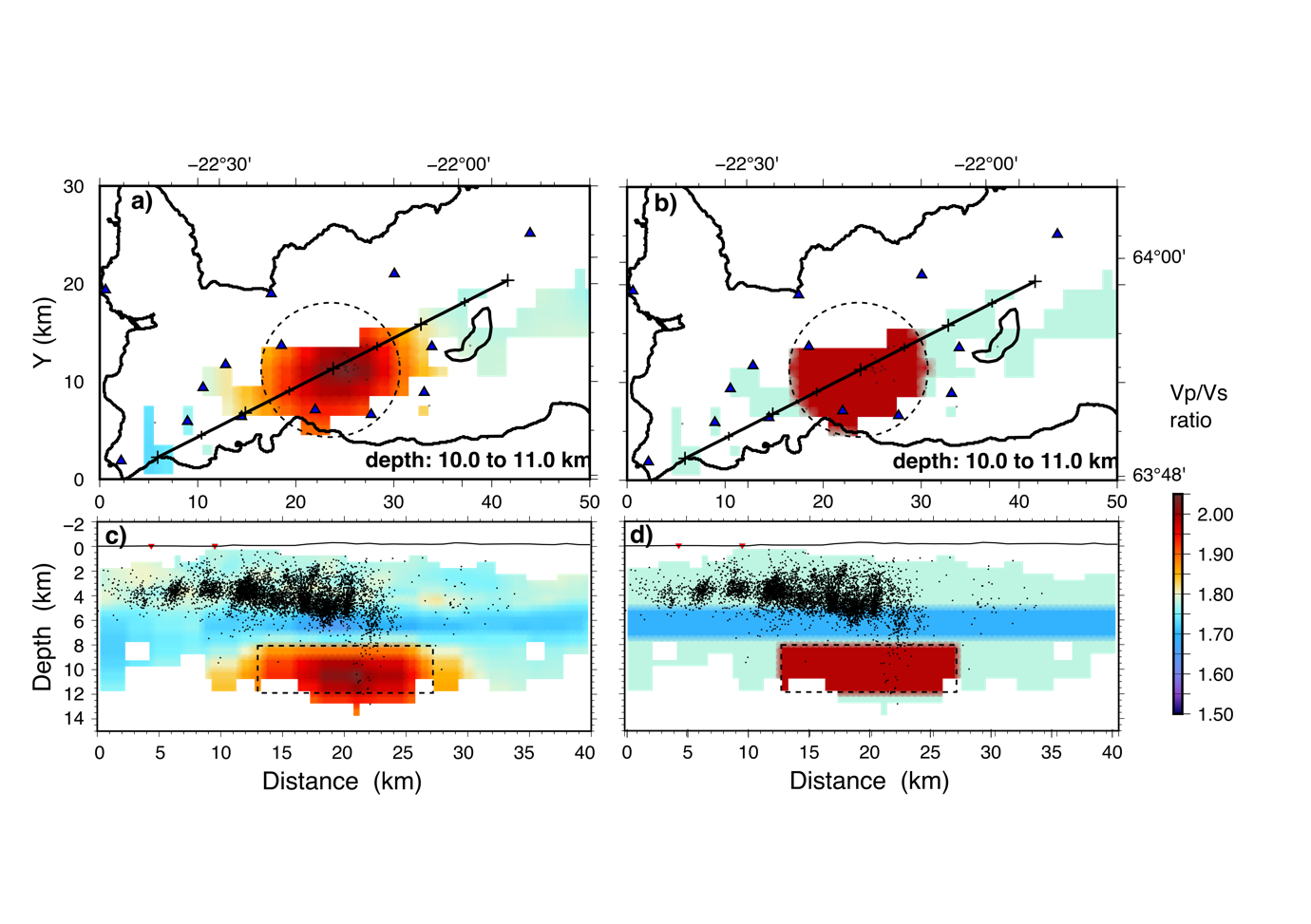


**Figure S6.** Same reconstruction test as in Fig. S5 except that the high Vp/Vs ratio anomaly is 14 km across. Map views of the reconstructed and true models in (a) and (b) respectively. Cross sections in panel (c) and (d). The width of the high Vp/Vs ratio anomaly is fairly well reconstructed, indicating that the true high Vp/Vs ratio anomaly imaged by the real data is likely narrower than this. The maps and cross sections were prepared with GMT version 5.4.6 (<https://www.generic-mapping-tools.org>) and Inkscape version 1.3.2 (<https://inkscape.org/>).


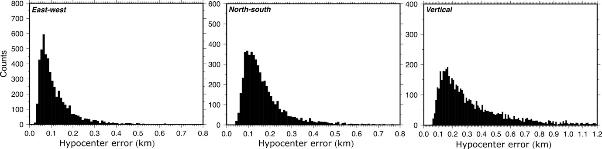


**Figure S7.** The hypocentral parameter uncertainties. Errors are larger for the depth parameter. 90% of the events have smaller depth uncertainty than 0.6 km. The histograms were prepared with GMT version 5.4.6 (<https://www.generic-mapping-tools.org>).

**
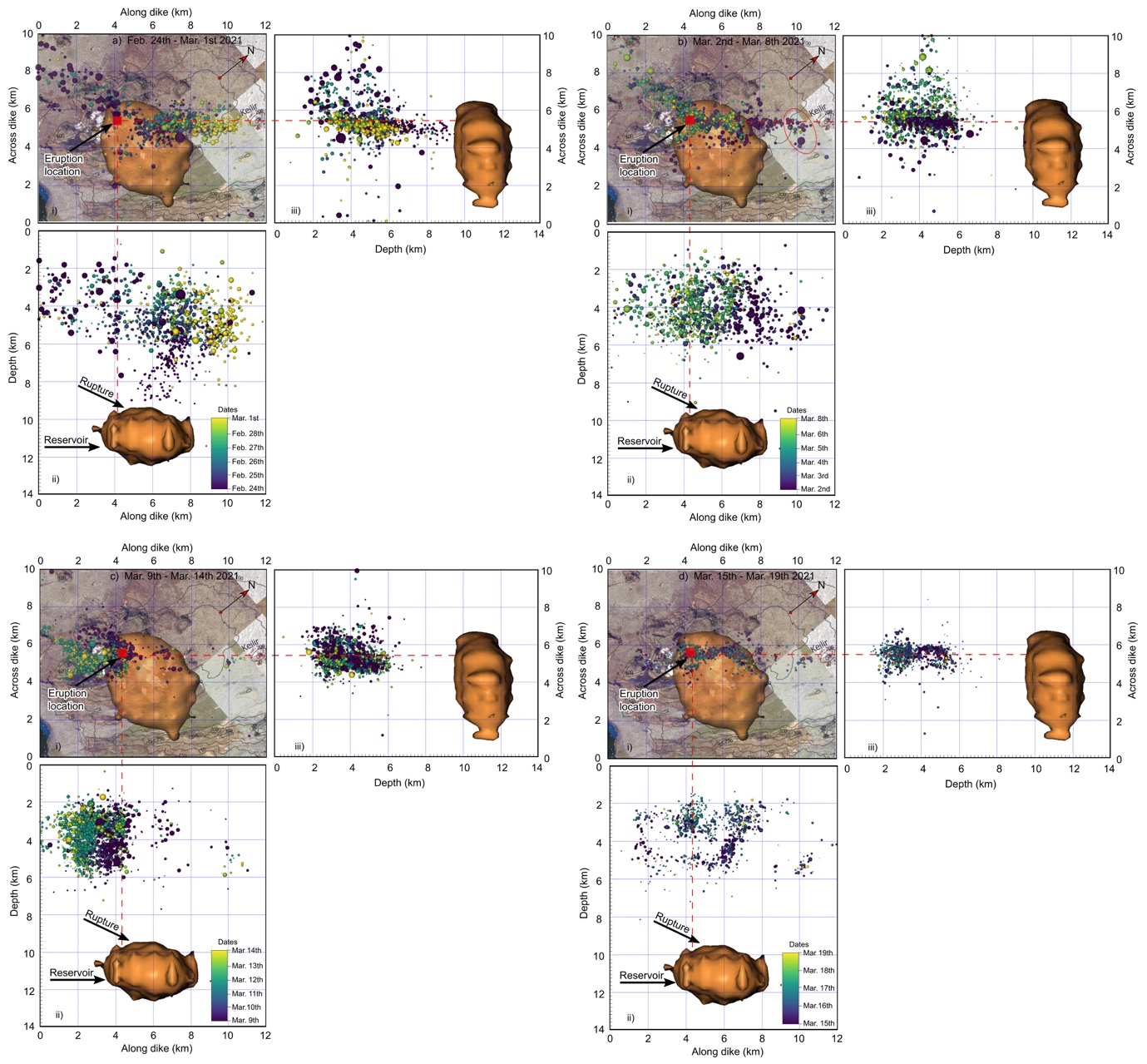
**

Maps Data: Google (c) 2023 Airbus, Maxar Technologies, CNES/Airbus.

**Figure S8.** Seismicity of the eruptive swarm in 2021 in four parts. (a) Initial rupture and dike-segment propagation to the northeast. (b) A set of events (red ellipse) suggest an E-W-striking fault, which may possibly have affected the dike propagation in this direction. The seismicity moved from this location to the southwest with much seismicity in the plate-boundary zone. (c) Dike-segment propagation to the southwest after passing the boundary zone. The seismicity here includes the two M>5 events on 14 March. (d) Seismicity indicates that the dike tries two possible paths/fingers to the surface, one of which reaches the surface to erupt. Size shows event magnitudes. Seismicity to the west is cut off to focus on the eruptive swarm. The brownish bubble is the top of the magma reservoir. The 3D images were generated by Voxler version 4 (<https://support/goldensoftware.com>), the maps on the plan view were downloaded from Google Earth Pro version 7.3.6.10201 (<https://earth.google.com>) and georeferenced in ArcGIS Pro version 3.0.36056 (<https://www.esri.com>), and the figure prepared in Inkscape version 1.3.1 (<https://inkscape.org>).

**
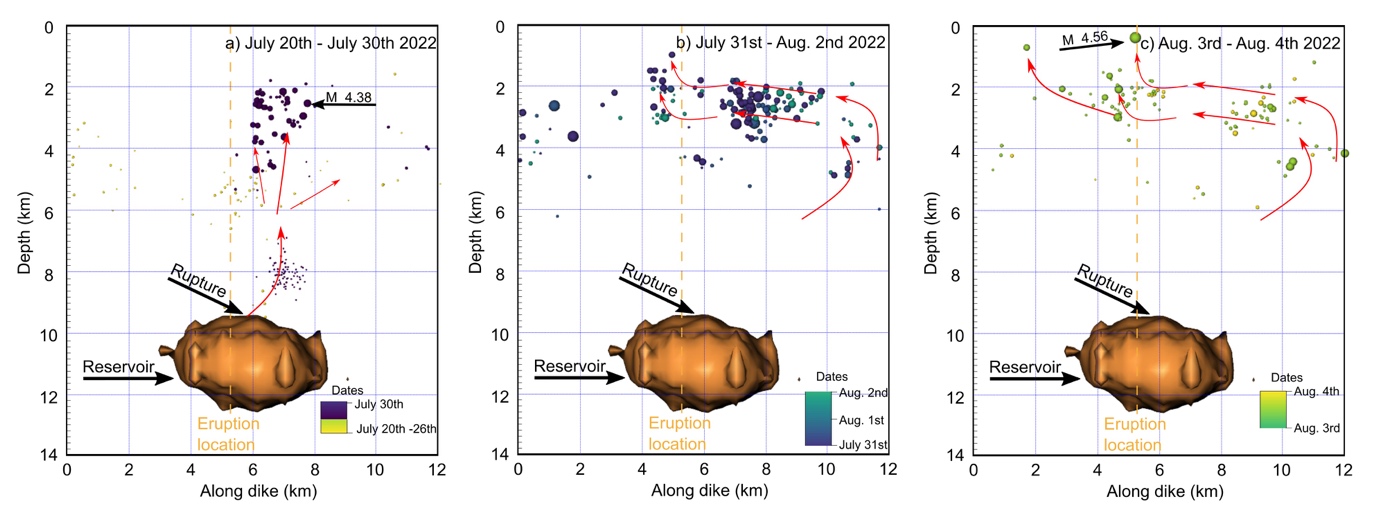
**

**Figure S9.** Seismicity of the eruptive swarm in 2022 and interpretation of magma flow direction (in the multiple dike initiated in 2021) in three parts. (a) Deep seismicity prior to 30 July was primarily below 8 km depth. At 8.30 am on 30 July seismicity began to migrate above 8 km depth and reached 2 km depth at about 5 pm the same day. (b) In the early morning of 31 July seismicity migrated 6 km southwest along the dike as the magma tried to find a path to the surface. (c) On 3 August a M4.6 earthquake occurred at a very shallow depth about a km southeast of the eruption site. The eruption started at 1:18pm the same day. The brownish bubble is the top of the magma reservoir. The 3D images were generated by Voxler version 4 (<https://support/goldensoftware.com>) and the figure prepared in Inkscape version 1.3.1 (<https://inkscape.org>).


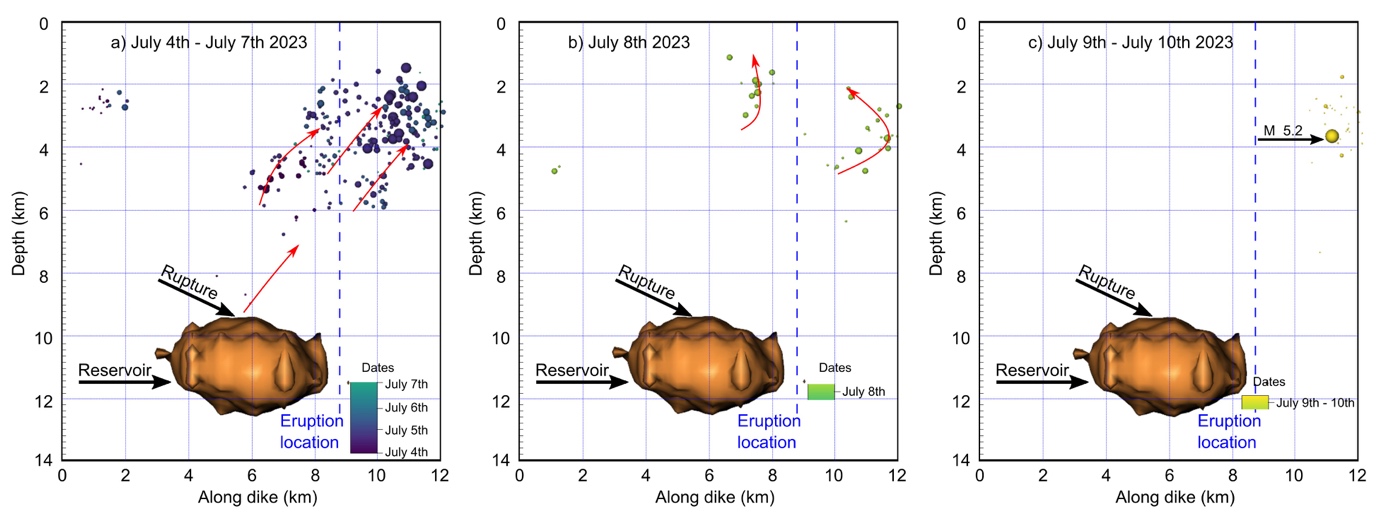


**Figure S10.** Seismicity of the eruptive swarm in 2023 and interpretation of magma flow direction (in the multiple dike initiated in 2021) in three parts. (a) There were relatively few deep events in 2023 compared with the those recorded during the 2021 and 2022 eruptions. However, in the afternoon of 4 July there were a few events in the roof of the reservoir. At 10 pm the same day seismicity occurred at 4-5 km depth. The following day seismicity migrated to 2 km depth. Seismicity between 2 and 4 km was mainly confined to the northeastern part of the dike. (b) Seismicity again picked up on 8 July, and (c) late on 9 July an M5.2 earthquake occurred close to the northeastern end of the dike. The eruption started at 4:40 pm on 10 July. The brownish bubble is the top of the magma reservoir. The 3D images were generated by Voxler version 4 (<https://support/goldensoftware.com>) and the figure prepared in Inkscape version 1.3.1 (<https://inkscape.org>).

**Figure S11.** a) Relocated events from January to March 2021. The square outlines the Fagradalsfjall area and shows the region for which events are selected in the lower panels. Background digital elevation model is the 2-m ÍslandsDEM from Landmælingar Íslands (National Land Survey of Iceland). b) Events in the Fagradalsfjall area plotted as time vs. depth. The blue arrow shows the start of the dike injection at about 10 km depth on the 24 Feb. The red arrow shows the start of the first eruption, 19 Mar, indicated by the shallow events. c) events during 24 Feb plotted as time vs depth. The red stippled line shows the estimated ascent rate of the magma (0.26 m/s). d) Same as panel c) for the second dike injection starting on 30 Jul 2022, and e) shows the same for the injection in 2023. The injection rate appears to be gradually slower, but on average about 0.2 m/s for the three eruptions. The map and time vs depth plots were prepared with GMT version 5.4.6 (<https://www.generic-mapping-tools.org>) and Inkscape version 1.3.2 (<https://inkscape.org/>).
